# Introducing the tablet-based Oxford Cognitive Screen-Plus (OCS-Plus) as an assessment tool for subtle cognitive impairments

**DOI:** 10.1038/s41598-021-87287-8

**Published:** 2021-04-12

**Authors:** Nele Demeyere, Marleen Haupt, Sam S. Webb, Lea Strobel, Elise T. Milosevich, Margaret J. Moore, Hayley Wright, Kathrin Finke, Mihaela D. Duta

**Affiliations:** 1 Department of Experimental Psychology, University of Oxford, New Radcliffe House, Radcliffe Observatory Quarter, Oxford, OX2 6AE UK; 2 Department of General and Experimental Psychology, Ludwig-Maximilians-Universität München, Munich, Germany; 3 Coventry University, Coventry, UK; 4 Hans-Berger Department of Neurology, Jena University Hospital, Jena, Germany; 5 Department of Computer Science, University of Oxford, Wolfson Building, Parks Road, Oxford, OX1 3QD UK

**Keywords:** Cognitive ageing, Diagnosis

## Abstract

Here, we present the Oxford Cognitive Screen-Plus, a computerised tablet-based screen designed to briefly assess domain-general cognition and provide more fine-grained measures of memory and executive function. The OCS-Plus was designed to sensitively screen for cognitive impairments and provide a differentiation between memory and executive deficits. The OCS-Plus contains 10 subtasks and requires on average 24 min to complete. In this study, 320 neurologically healthy ageing participants (age *M* = 62.66*,* SD = 13.75) from three sites completed the OCS-Plus. The convergent validity of this assessment was established in comparison to the ACE-R, CERAD and Rey–Osterrieth. Divergent validity was established through comparison with the BDI and tests measuring divergent cognitive domains. Internal consistency of each subtask was evaluated, and test–retest reliability was determined. We established the normative impairment cut-offs for each of the subtasks. Predicted convergent and divergent validity was found, high internal consistency for most measures was also found with the exception of restricted range tasks, as well as strong test–retest reliability, which provided evidence of test stability. Further research demonstrating the use and validity of the OCS-Plus in various clinical populations is required. The OCS-Plus is presented as a standardised cognitive assessment tool, normed and validated in a sample of neurologically healthy participants. The OCS-Plus will be available as an Android App and provides an automated report of domain-general cognitive impairments in executive attention and memory.

## Introduction

One of the key challenges in assessing cognitive dysfunction is to detect not only obvious impairment, but to also pick up on subtle impairments in different cognitive domains. Traditionally used global screening tools for cognition, such as the Mini-Mental State Examination (MMSE^1^) and the Montreal Cognitive Assessment (MoCA^2^), rely on a summated score from subtasks with a single cut-off value for obvious impairment, irrespective of age. Sometimes a broad-brush correction for education level is made by slightly adjusting the cut-off value. While item response theory analyses have been applied to these assessment tools, in every-day practice they still take a binary approach to cognition by relying on a sum score.

Consequently, the screens are unable to detect subtle or domain-specific impairments due to the lack of subtask normative data and, frequently, the lack of population specific normative subtask cut-offs^3,4^. In addition, the MMSE and MoCA contain many subtasks which are meant to assess non-language cognitive functions but are heavily dependent on intact language function. For example, the MoCA’s attention subtask requires participants to verbally repeat sequences of numbers^2^. Patients with a language deficit would appear to be impaired on this task, regardless of their underlying attentional capacity. This inability to separate cognitive impairments is problematic for patient populations characterized by language impairments, such as some patients with stroke and dementia^5^. Similarly, the language component makes the screens less appropriate in populations with low literacy^6–9^. The level of language requirements may thus cause interpretation problems and lead to suboptimal tests.

The Oxford Cognitive Screen-Plus (OCS-Plus) aims to avoid undue loading of language requirements by emphasizing visual-oriented assessments. This tablet-based cognitive assessment tool is a follow-up of the paper-based OCS^10^ and was designed to be equally inclusive. The OCS is a validated and normed standardized paper-based test that provides a domain-specific cognitive profile for stroke survivors. It covers five core cognitive domains (attention, language, memory, number and praxis) and includes many aphasia- and neglect-friendly subtasks, i.e., through usage of high frequency words and central presentation of items. Recently, the OCS has been shown to be more sensitive to cognitive deficits in acute post-stroke cohorts than both the MoCA and MMSE^7,11^. While this approach is ideal for acute stroke settings, a more sensitive and detailed assessment is required to detect more subtle domain-general cognitive impairments in this and other populations. This is why we developed the OCS-Plus.

An important and novel aspect of the OCS-Plus is that it is a digital tool, ideal for computer tablet-based assessment. The widespread adoption of tablet computers has facilitated cost-effective computerized cognitive assessment tools^12–14^. Computerized cognitive assessments present several important advantages over pencil-and-paper-based assessments, including the standardization of test administration, recording of more detailed response metrics, and automated scoring^15,16^. In addition, Miller and Barr^17^ called for tools with automated scoring and reporting to reduce the potential for scoring and data entry errors, and to facilitate real-time evaluation of standardized performance. This is implemented in OCS-Plus. Therefore, the test does not require the presence of a neuropsychologist but can be administered by any clinically trained staff following the manual and brief instructional video. The described computerization facilitates the test administration by removing various constraints. In particular, the ability for the test to be conducted at home, or in a quiet, remote clinic setting, removes the need for participants to travel to specialized centers. This could offer a potentially higher safety and/or more accessible setting, compared to centrally based hospitals or health centers, and could provide a critical step towards telemedical neuropsychological assessment.

A previous iteration of the OCS-Plus was translated and validated in a population of older adults in a low literacy and socioeconomic setting^18^. The results of that study indicated that the OCS-Plus showed high task compliance and good validity, improving the measurement of cognition with minimal language content, thereby avoiding floor and ceiling effects present in other short cognitive assessment tools.

The purpose of the current study is to present the test, describe the tasks, report standardised normative data, and investigate the validity, internal consistency, and reliability of the OCS-Plus within a group of neurologically healthy older adults from a pooled English and German normative sample.

This psychometric validation is a necessary first step in order to determine whether the OCS-Plus represents a useful method for detecting and differentiating between a range of subtle cognitive impairments. For this, we would anticipate seeing a range of performance in healthy ageing, demonstrating sensitivity to these expected demographic variables, similar to what was found in the large epidemiological validation of OCS-Plus in rural South Africa^18^. We envision that the OCS-Plus can be applied in a multitude of healthy and pathologically aging populations, encompassing various neuro-degenerative diseases, acquired brain injuries, viral infections affecting brain function, psychiatric conditions and broad cardiovascular factors including diabetes, hypertension, obesity, and smoking^19^. A validation of the OCS-Plus in patient populations is beyond the scope of the present paper and will require future studies. The aim of this paper is to build a basis for these clinical cohort studies by reporting an initial investigation of the properties of the OCS-Plus in healthy adults.

## Methods

### Participants

A cohort of 320 neurologically healthy participants completed the OCS-Plus (for sample sizes by subtest, see the normative tables). Participants were recruited in three different sites: Oxfordshire, UK (n = 161), Coventry, UK (n = 73) and Munich, Germany (n = 86), and were all of white European ethnicity. All participants were recruited through convenience sampling from existing participant databases at each site. Original recruitment to the databases was primarily from contacting next of kin to patients with stroke or dementia screened for research at each site, those who contacted a researcher through our websites, or individuals who signed up for research at open days or during educational courses for senior citizens. For the UK cohort, participants were included in this investigation if they were: 18 years of age or older; had no self-reported neurological or psychiatric condition; able to stay alert for more than 15 min; and able to speak fluent enough English to comprehend task instructions. In the German cohort, all participants were native German speakers, had no self-reported neurological or psychiatric condition, and completed a cognitive screening, i.e., MMSE, on the same day as the OCS-Plus assessment. None of the participants were excluded due to demonstrating any evidence of cognitive impairment on the MMSE, or for any other reason.

Standard education was differentially characterized for the German and UK samples due to variations in education scoring, whereby the German cohort was marked on school years (*M* = 11.29, SD = 1.86), and the UK cohorts were marked on number of years in formal education including higher education (*M* = 16.02, SD = 3.94). To harmonise this, for the UK cohort on the basis of school running from 5–18, we classed standard education as ≤ 12 years and higher education as ≥ 13 years. For the German cohort, in the same way the education was binarized to differentiate between having further education past 18 (high education) or not.

### Procedure

All participants provided written informed consent under local ethics (Oxford University ethics reference ‘MSD-IDREC-C1-2013-209’; Coventry University ethics reference ‘P33179’ approved by Coventry University Research Ethics Committee (internally funded by Coventry University Pump-Prime Research Grant Scheme); Ludwig Maximilian University of Munich psychology department ethics committee reference ‘10_2015_Finke_b’). Participant recruitment and procedures were in line with the Helsinki declaration. All participants were invited to the departments at the Universities of Oxford, Coventry, and Munich and were assessed by trained PhD students and Research Assistants, under supervision of the respective group leaders who are experienced neuropsychologists. All participants were seated in a quiet room with the tablet placed on a table between them and the experimenter. All participants completed the OCS-Plus in a single session.

The demographic information for the complete cohort of participants is presented in Table 1, and raw age and education in years distributions are visualised in Supplementary Material Figure S1. For German participants, the OCS-Plus and all other neuropsychological tests were administered in German.

**Table 1:** Demographic characteristics of the normative sample and subdivided demographics for UK and German samples on OCS-Plus subtasks.

Characteristic	Overall		UK		German	
	*N*	Observed	*n*	Observed	*N*	Observed
Age	320	62.66 (13.75, 23–99)	234	60.51 (14.91, 23–99)	86	68.49 (7.26, 50–81)
Education	316	84:232	230	51:179	86	33:53
Hand	318	296:19:3	232	211:18:3	86	85:01:0
Sex	320	176:144	234	128:106	86	48:38

Oxford Cognitive Screen-Plus (OCS-Plus). Age is formatted as follows: *M *(SD, range), education is formatted as follows: standard education/higher education. Hand refers to dominant hand of the participant throughout their life and is formatted as right:left:ambidextrous. Sex is binarised as male or female based on presentation of the individual at testing as coded as male:female. Some values are missing due to attrition over time (2014–2019), with some participants no longer available to be contacted for correct information. Standard education was differentially characterized for the German and UK samples due to variations in education scoring, whereby the German cohort were marked on school years, and the UK were marked on number of years in formal education including higher education. For this reason, we do not present education in years as the differences between German and UK samples would be misleading to the idea that the German sample were less educated. For the UK cohort on the basis of school running from 5–18, we classed standard education as ≤ 12 years and higher education as ≥ 13 years. For the German cohort having a university degree separated the standard or high education sample. No data were collected on ethnicity.

By combining the cohorts, we provide a sample of adults across the lifespan, primarily focused on older adults. Prior to combination, the subsamples' potential difference in scores was evaluated by comparing performance on each of the OCS-Plus subtasks between groups using Bonferroni correction for multiple comparisons. Participants did not perform statistically different on any OCS-Plus subtasks, with the exception of the Figure Copy test, in which the UK cohort was found to perform significantly better (Mann–Whitney test, German [*n* = 86] mean = 54.16, UK [*n* = 229] mean = 55.72, *U* = 6779.5, *p* < 0.001), although the difference was small. On the basis of having a larger pool to garner potential task cut offs from, only data from the larger UK cohort was used for the Figure Copy test. For full details on the comparisons between the UK and German cohorts see Supplementary Tables S1–S3. It must be noted that prior to correction for multiple comparisons, there were other statistically significant differences. However, these did not affect the majority task-specific cut offs, or where they did the difference was marginal and we believe these differences do not justify separating the groups further in order to generate separate clinical cut offs.

### OCS-Plus

The OCS-Plus comprises ten short tests and can typically be completed in under 25 min. The validation of the tool was completed using a stand-alone application on Windows Surface Pro tablets developed using Matlab^20^ and Psychophysics Toolbox^21–23^. The OCS-Plus tool has now been developed on an Android platform with data either locally removed at end of session, or uploaded to a cloud server, dependent on user settings. This Android version creates an automated report comparing performance to the normative data presented here. For access to the tool, please contact the Oxford University Innovations Health Outcomes team.

A brief description of each task, the cognitive functions they aim to assess, and the order of administration is provided in Table 2. In addition, a video demonstration of each of the tasks as well as a full run-through of the OCS-Plus with a control participant is available to view online^24^. After each task, the examiner documents the condition of testing to flag any potential confounds, such as task interruptions or participant fatigue. Similarly, when a subtask is skipped, the reason for why the task was not attempted is recorded. This extra information subsequently aids the interpretation of the performance and report.

**Table 2:** Brief subtask descriptions and order of administration within OCS-Plus.

Task order	Task name	Brief description	Scoring
1	Picture naming	The Picture Naming task assesses visual object recognition and access to semantic/conceptual knowledge about the objects, word finding, and articulation. There are four low frequency items to name. Performance is relatable to language as well as reported under the memory domain (word finding). These items also form part of the incidental Episodic memory test further in the OCS-Plus.	Four items are scored for correct response giving a score range from 0–4
2	Semantics	The Semantics task assesses both specific object and semantic category knowledge. This uses multiple choice matching within semantic categories of exemplar pictures to names. Performance is relatable to language as well as memory domain. These items also form part of the incidental Episodic memory test further in the OCS-Plus.	Four items are scored for correct response giving a score range from 0–4
3	Orientation	The Orientation task assesses orientation in time and space, related to long term memory. The participant is asked which year, month and date it is, and is then also asked whether they can name the current prime minister.	Four questions are scored for correct response giving a score range from 0–4
4	Word memory encoding	The Word Memory Encoding task assesses encoding of 5 words over 2 attempts. The participant is given a list of five words to remember (bicycle, mist, wardrobe, teacher, and rectangle). The participant is asked to recall the items immediately after presentation, then, regardless of performance, the participant is presented with the word list again and asked for immediate recall a final time. This forms immediate verbal recall over two stages of encoding.	Five words are scored for correct response giving a score range from 0–5, encoding is scored separately for first immediate recall and second immediate recall
5	Trails	The Trails task assesses trail making and set switching. The task has three components. There are two baseline components: (i) connecting circles in decreasing order of size, in the presence of square distractors, and (ii) connecting squares in increasing order of size, in the presence of circle distractors. These baselines are compared with a complex set switching condition in which participants draw a trail alternating between circles and squares, with circles going in descending order of size and squares in ascending order of size. The items are positioned pseudo-randomly, so that a correct trail can be drawn without going through any of the other shapes, and the items appear in a central section on the page. Performance is timed.	Baseline squares and circles trails are both scored for total correct connections out of seven
		The effect of switching is assessed by the proportion of the switching task over performance on both baseline tasks summed.	Processing speed was calculated as the sum time taken on both versions of the baseline tasks (circles or squares) divided by proportional accuracy. This correction attempts to account for speed/accuracy trade-offs (i.e., will correct very fast processing speeds where a participant only connected a few shapes)
6	Verbal recall	The Verbal Recall task assesses the recall of the 5 encoded words. The participant is asked to reproduce the list of words they memorised in the Word Memory Encoding task. If the participant is unable to recall all words correctly, multiple choice recognition options are given for each missed or incorrectly recalled target word. In the recognition part, four vertically distributed options are shown and read to the participant and they are asked to make a forced choice response.	Five words are scored for correct free recall giving a score range from 0–5 for recall and 0–5 for recall and recognition total
7	Episodic recall	The Episodic Recall task assesses incidental recognition of items and tasks which the participant experienced earlier in the assessment. Four multiple choice questions address the participant’s recognition of previously presented items, pictures, and words.	Four items are scored for correct response giving a score range from 0–4
8	Figure copy/recall	The Figure Copy/Recall task assesses constructional praxis and non-verbal memory. The participant is asked to copy a composite image of geometric shapes, with the image to be copied initially present at the top of the page for the duration of the copying. Subsequently, and immediately after, the participant is asked to draw the same composite figure once more, this time from memory.	Scores are given per specified element (20) drawn in terms of: presence, position, and overall accuracy. Each element gets a score out of 3 and therefore the total possible score is 60 for both Figure Copy and Figure Recall
9	Rule finding	The Rule finding and Switching task assesses executive functioning through problem solving and goal attainment as well as flexibility in switching and adapting to new rules. The participant is presented with three columns of alternating geometric shapes (squares-triangles-squares), rows of alternating luminosity (dark–light), and a red dot that moves around the pattern following certain rules. The participant has to try and pick up the rules to predict where the dot is going to go next based on previous moves (the most recent preceding position is highlighted on the display). The rules will change throughout the test without giving any notice and it is the participant’s task to pick up on the change and work out the new rule. There are five rules in this test.	The Rulefinding task produces a score for overall accuracy and for number of rules detected. The total number of correct anticipations, excluding those immediately after a rule change, give a total accuracy score out of 46
			If at least two consecutive guesses are correct within the same rule then the rule is scored as correctly detected. There are five rules and the score thus ranges 0–5
10	Cancellation	The Cancellation task assesses selective attention and spatial working memory. The cancellation task in OCS-Plus is a search task including semantic category items, assessing organized search. The participant is asked to select drawings of fruit amongst drawings of common fruit and vegetables immediately followed with an invisible version of the same cancellation display. In the version administered first (“feedback version”) the selected drawings are framed, and this visual feedback stays on the screen for the duration of the test. In the invisible cancellation version administered second (“no feedback version”) the visual feedback is only visible for the duration of the pen tap and it disappears afterwards; this requires the participants to remember the locations and items previously selected and inhibit revisiting those.	Total number of correct fruit selections in both the visible and invisible feedback conditions provide a score range of 0–30
			False positives are scored as 1 error point per incorrect item selected in the visible cancellations task
			Correct revisits are scored as 1 error point per time an item is reselected in the invisible feedback conditions

Oxford Cognitive Screen-Plus (OCS-Plus). Images of each subtask are presented in Supplementary Material. A video demonstration of all tasks is available on Open Science Framework^24^.

The OCS-Plus uses accuracy-based measurements where possible. This approach differs from other conventional neuropsychological assessments which use response time to quantify performance. A time-based scoring method generally assumes that healthy controls perform at ceiling, and this assumption does not always hold true^25^. Additionally, relying on time-based performance metrics is problematic for clinical populations containing participants who may exhibit response slowness for physical reasons, (e.g. motor weakness or muscle conductance) which may confound assessment of underlying cognitive deficits^26^**.** It has also been suggested that older populations prioritize slower, more controlled performance over speed-based response strategies^27^. For these reasons, the OCS-Plus employs accuracy-based outcome measures rather than response time-based metrics wherever appropriate. One exception to this approach is the OCS-Plus’ measurement of processing speed, which is inherently time-based. However, this measure still takes accuracy into account and is derived by dividing time taken by task accuracy. This proportional scoring method means that patients with slow performance and low task accuracy will be flagged as exhibiting abnormally slow processing speeds.

**Figure 1:**
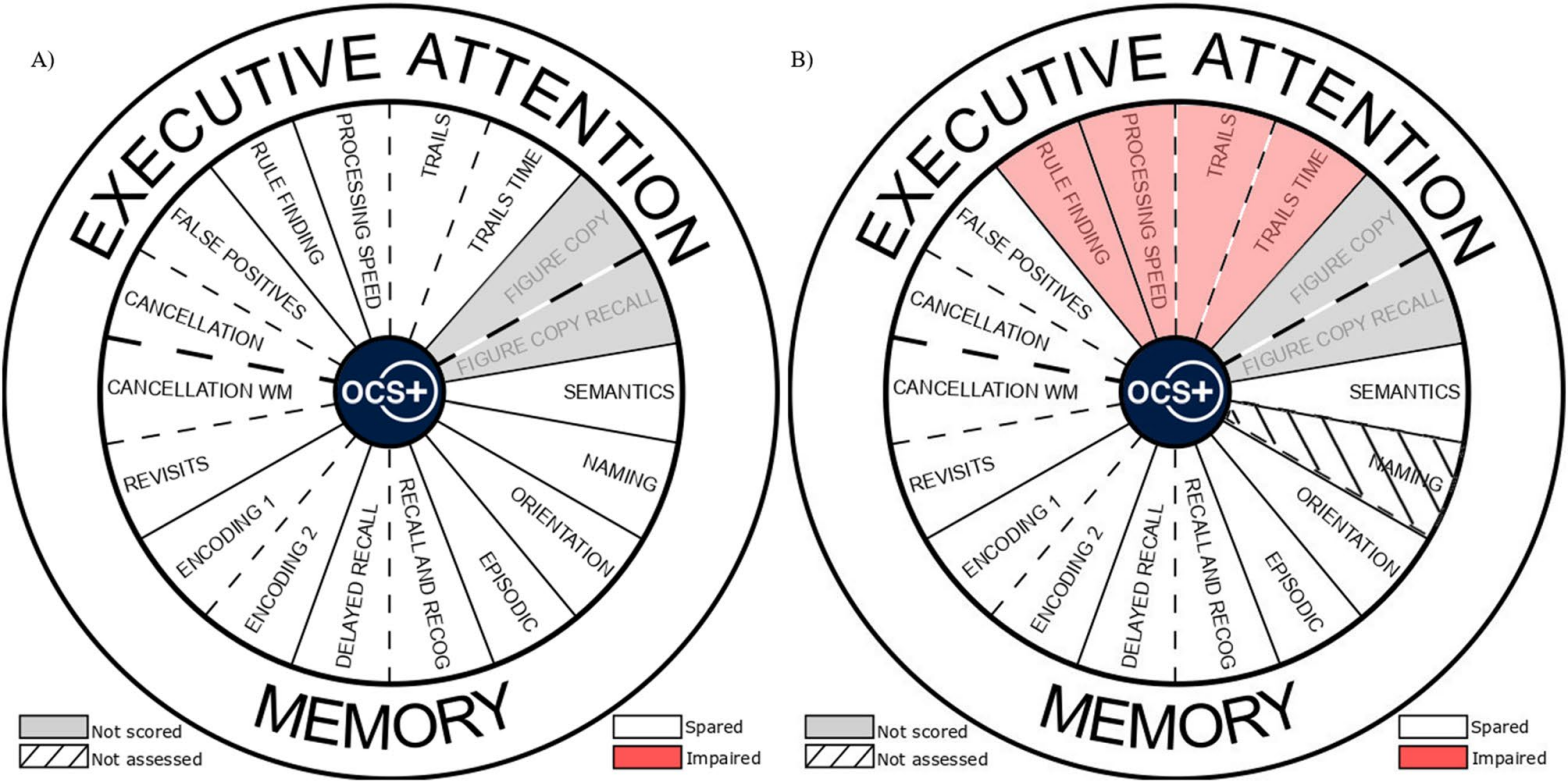
Part (**A**) represents the base report outcome wheel from the Oxford Cognitive Screen-Plus which is edited depending on performance of the participants (i.e., impaired or spared compared to norms), and (**B**) Represents a mockup of a patient who was impaired in the Trails and Rule Finding tasks compared to age matched normative data and who was not assessed with the picture naming task. Figure Copy and Figure Copy Recall are grey due to not currently having automatic scoring implemented in the app. Figure created in Inkscape (version 1.0.2, https://inkscape.org/) available at https://osf.io/ajzht/ under a CC-BY4.0 license.

All tests were designed to have low educational and language demands by using demonstrations and practice trials, short-high frequency words, and multimodal presentations. The OCS-Plus has previously been validated in low-literacy and low-education groups in South Africa and demonstrates good usability (see^18^). In addition, the design of the OCS-Plus includes an integrated code for translation and adaptation to other languages. At the end of the session, the newly developed OCS-Plus android app automatically produces an in-app report per task with clear indications of whether the participant is impaired compared to age-categorised normative data using a visual summary of the task and domain impairments (see Fig. 1). This visualisation is similar to the OCS visual snapshot result^10^. Data presented in this paper is from the original Matlab version of the app, the Android app uses the same tasks, stimuli, and instructions and though we expect no differences in performance, further application-specific data is planned.

### Convergent and divergent validity

The OCS-Plus was validated by comparing specific subtasks to a series of analogous standardised neuropsychological tests in order to provide measures of convergent and divergent validity. Convergent validity of the OCS-Plus subtasks was measured against specific existing neuropsychological standardized tasks assessing the same underlying cognitive domain construct. Divergent validity of OCS-Plus was established by comparing the tasks both to a non-cognitive construct in a mood measurement as well as to different cognitive constructs as measured by contrasting cognitive domain assessments. See Supplementary Figures S2–S4 and Table S5 for a summary of the specific comparisons which were conducted and graphs per correlation.

#### Addenbrooke’s Cognitive Examination Revised (ACE-R)

The ACE-R^28^ is a short screening test designed to detect dementia-related cognitive impairment. The ACE-R was developed following the MMSE, which it incorporates, and requires approximately 20 min to complete. Performance on the ACE-R is quantified using a single total score out of 100 points (p) which is calculated by summing subtask scores across five domains: orientation and attention (18p), memory (26p), verbal fluency (14p), language (26p), and visuospatial processing (16p).

#### Consortium to Establish a Registry for Alzheimer's Disease (CERAD)^29^

The CERAD-Plus test battery^30^ measures cognitive performance in domains which are specifically impaired in Alzheimer’s disease patients: memory, language, praxis, and orientation. This screening tool is able to differentiate between patients with mild and severe impairments and is therefore particularly useful for quantifying impairment severity and documenting the progression of cognitive decline over time. Furthermore, the CERAD-Plus has been found to have good objectivity, reliability, and validity, and has been translated in numerous languages^29–31^.

The CERAD-Plus contains semantic and phonemic verbal fluency tasks^32,33^, the abbreviated Boston Naming Task (BNT^34^), the MMSE^1^, the Word List Task (50p^35–37^), a visuospatial constructional praxis task, and the Trail Making Test (TMT^38^). These subtasks are designed to assess a wide range of cognitive abilities including word retrieval, recognition, immediate and delayed recall, production, processing speed, cognitive flexibility, and executive function. However, this battery does not formally assess attention, though the TMT contains some attentional aspects^39^. Each CERAD-Plus subtask has been individually normed. This battery requires approximately 30–45 min to complete.

#### Rey–Osterrieth Complex Figure Test (ROCF)

The ROCF is a visuospatial praxis test that draws upon various cognitive functions, including attention, visuospatial abilities, non-verbal memory, and task planning skills^40^. This task has three conditions: copy, immediate recall, and delayed recall. In the copy condition, participants are presented with a complex line drawing and are asked to draw a copy of this figure from sight. In the immediate recall condition, the reference figure is removed, and participants are immediately instructed to draw the figure from memory. Finally, in the delayed recall condition, participants are asked to reproduce the figure from memory after a 30-min delay period. Performance on the ROCF is scored according to the quantitative scoring system of Meyers and Meyers^41^, which includes 18 distinct figure elements which are separately scored with 0 to 2 points depending on correctness of position and completeness. Each figure reproduction is given a total score out of 36 possible points. This investigation only employs the copy and immediate recall conditions, as these conditions are most comparable with the OCS-Plus Praxis subtask. Participants are assigned a ROCF proportional score denoting the memory score as a percentage of the copy condition score for comparison with the OCS-Plus Figure Copy Recall score.

#### The Star Cancellation Test

The Star Cancellation Test is a visuospatial scanning task and part of the Behavioural Inattention Test^42^, a screening battery designed to assess the extent of hemispatial neglect. This task consists of a pseudorandom search array of small and large stars, letters, and short words presented across a landscape A4 sheet of paper. Participants are instructed to search through this matrix and identify all small stars while ignoring all distractor stimuli. Participants are allowed 5 min to complete this task. Each participant is given a total score out of 56, representing the number of targets successfully identified.

#### Beck’s Depression Inventory (BDI)

The BDI^43^ is a standardized, self-report questionnaire that aims to assess the presence and severity of depression symptoms. In this questionnaire, participants are presented with a series of 21 Likert scale statements. Overall performance is scored by summing participant’s Likert scale responses into a total score out of 63, with higher total scores representing a higher level of depressive symptoms. This measure was used to establish divergent validity of the OCS-Plus subtasks, where this non-cognitive construct should not be highly correlated with the specific cognitive constructs underlying OCS-Plus sub-tasks.

### Planned analysis

The impairment threshold for each individual OCS-Plus subtask was calculated based on the score distributions present within the healthy ageing control participant group. For subtasks with a restricted range of possible subtask scores, 5th and 95th percentile-based impairment thresholds based on uncorrected sample score distributions were used. For all other subtasks, cut-offs at ± 1.65 SDs control mean were employed, following standard practice in neuropsychological testing^44^. Next, the reliability and validity of performance on the OCS-Plus subtasks were evaluated. Task-specific correlations with established standardized measures were performed to provide evidence for convergent and divergent validity. There are no gold standard criteria for convergent validity measures, aside from expecting “high correlations”^45^. Several established tests report convergent validities ranging as low as − 0.19 (e.g.^46^). As such, we will interpret correlations to be significant where we have 80% power to detect. For our validation, with an achieved sample size of between 85–159 per correlation, an alpha of 0.05, power of 80%, we could detect correlations no smaller than 0.19 (or − 0.19) to 0.26 (or − 0.26). In line with previous work and in line with our statistical power, we therefore would validly interpret correlation coefficients between the OCS-Plus and external measures above 0.19 (or below **− **0.19). In line with other studies validating cognitive tests, which have proven clinically relevant, we defined correlation coefficients exceeding 0.20 as acceptable and relevant.

Internal consistency was evaluated using Cronbach’s alpha as a measure of single factor internal reliability. In addition, some of the participants took part in additional projects so that we could use their data to get first insights into the reliability of OCS-Plus testing over time. Importantly, it should be noted that due to the use of opportunity data we are analyzing a wide range of inter-test intervals. Test–retest validity was determined using Wilcoxon signed rank test with continuity correction. Finally, we present one theoretically based potential methodology for cognitive domain scores, which could be used to facilitate data interpretation within clinical settings.

All analyses were performed in R^47^ version 3.5.1 (https://www.R-project.org/) using packages such as *readxl*^48^ version 1.3.1 (https://readxl.tidyverse.org/), *dplyr*^49^ version 0.8.3 (https://dplyr.tidyverse.org/) for data manipulation, *ggplot2*^50^ version 3.3.2 (http://ggplot2.org) for plotting data, *rcompanion*^51^ version 2.3.7 (https://rcompanion.org/handbook/) for computing Wilcoxon effect sizes, *sjstats*^52^ version 0.17.8 (https://strengejacke.github.io/sjstats/) for eta effect size calculations. The underlying raw data, the codebook containing distribution statistics on all variables, and the code used for all analyses reported are openly available through the Open Science Framework^53^.

## Results

### Normative data

The average time taken in minutes between starting the Picture Naming task and finishing the Cancellation task was *M* = 23.88, SD = 5.78, range = 13.72–57.29. The normative data of OCS-Plus subtasks and proposed cut-offs for impairment based on the full sample can be found in Table 3. Individuals who took longer than average, primarily did so due to taking breaks after recall tasks or at the end of sub-tasks (we ensured these breaks did not come in between encoding and recall phases). In a few cases there were longer sessions due to technical issues with the tablet, such as battery or updating issues etc.

**Table 3:** Data and cut offs for impairment (z-scores greater than 1.65 SD from the mean or scores lower than 5th centile) for OCS-Plus subtasks.

Task name	Measure	*N*	*M*	SD	Median	Min	Max	Cut-off
Picture Naming	Overall accuracy	320			4	1	4	< 3
Semantics	Overall accuracy	320			4	2	4	< 3
Orientation	Overall accuracy	320			4	2	4	< 3
Word recall	Encoding 1	320			5	1	5	< 3
	Encoding 2	320			5	0	5	< 4
	Delayed recall accuracy	320			4	0	5	< 2
	Delayed recall and recognition	320			5	3	5	< 4
Episodic recognition	Episodic recall accuracy	319			4	1	4	< 3
Trails	Processing speed	320	33.83	18.70		11.02	143.38	> 64.69
	Executive score	320	81.68	25.04		0	100	< 40.54
Rule finding	Overall accuracy	320	26.74	8.20		3	43	< 13.21
	Number of rules learned	320	2.99	1.32		0	5	≤ 1
Figure copy	Figure copy accuracy	229*	55.72	6.67		19	60	< 44.41
	Figure recall accuracy	315	43.64	10.56		13	60	< 26.22
Cancellation	Overall accuracy	317	29.67	.63		27	30	< 28.63
	False positives	317			0	0	1	≥ 1
Invisible cancellation	Overall accuracy	318	28.44	1.82		13	30	< 25.44
	Correct revisits	318			0	0	10	≥ 1

Oxford Cognitive Screen-Plus (OCS-Plus). Means and SDs reported only for tasks with sufficient range in values. We proposed to use Z-score based impairments greater than 1.65 SDs from the mean for tasks with larger ranges of possible scores (measures from Trails, Rule finding and Figure copy tasks, as well as Cancellation and Invisible Cancellation accuracy). Measures with small ranges of possible scores are reported as median, and 5th centiles are chosen from determining cut-offs for impairment (Picture Naming, Orientation, Semantics, Word Recall, False positives, and correct revisits). Centiles for number of rules learned, false positives, and correct revisits were conservatively increased to greater than or equal to one as the centile was 0 on each measure. Asterisks reflect UK only normative data.

### Trends of performance across age and education

Cognitive abilities are not uniform across all age and education groups^54^. For this reason, the normative data should be split into subgroups and education- and age-specific impairment thresholds established. Based on standard neuropsychological approaches, and in order to have age groups which have large and approximately equal sample sizes, the sample was split into three age groups: below 60, 60–70, and above 70, following a similar and successful grouping strategy in the original Oxford Cognitive Screen^10^.

These age groups were chosen to both fit in with the classifications of the Oxford Cognitive Screen for cross-screen comparison, but also to ensure we had approximately equal age groups. By splitting the groups as we have, the age-adjusted cut-offs based on equal group sizes, ensuring more reliable age-adjusted cut-offs.

Several significant differences in performance were identified between various age groups before correction for multiple comparisons, highlighting the need for age-specific impairment thresholds on the OCS-Plus subtasks, which are provided in Tables 4 and 5.

**Table 4:** Data and cut offs for centile-based impairment per age category (scores lower than 5th centile) for each OCS-Plus subtask.

Task	Measure	Aged < 60					Aged > 60 and < 70					Aged > 70				
		*n*	Med	Min	Max	Cut-off	*n*	Med	Min	Max	Cut-off	*n*	Med	Min	Max	Cut-off
Picture naming	Accuracy	111	4	1	4	< 3	101	4	3	4	< 3	108	4	2	4	< 3
Semantics	Accuracy	111	4	2	4	< 3	101	4	2	4	< 3	108	4	2	4	< 3
Orientation	Accuracy	111	4	3	4	< 4	101	4	3	4	< 3	108	4	2	4	< 3
Word recall	Encoding 1 accuracy	111	5	2	5	< 3	101	5	1	5	< 3	108	4	2	5	< 2
	Encoding 2 accuracy	111	5	4	5	< 5	101	5	0	5	< 4	108	5	3	5	< 4
	Delayed Recall accuracy	111	5	0	5	< 3	101	4	0	5	< 2	108	4	0	5	< 1
	Delayed Recall and recognition accuracy	111	5	4	5	< 4	101	5	4	5	< 4	108	5	3	5	< 4
Episodic Recognition	Episodic accuracy	110	4	1	4	< 3	101	4	2	4	< 3	108	4	2	4	< 3
Cancellation	False positives	111	0	0	1	≥ 1	99	0	0	1	≥ 1	107	0	0	1	≥ 1
Invisible cancellation	Correct revisits	110	0	0	4	≥ 1	100	0	0	6	≥ 1	108	0	0	10	≥ 1

Oxford Cognitive Screen-Plus (OCS-Plus). *Cut-off* refers to 5th centile for accuracy data and 95th centile for error data (i.e., false positives and correct revisits). Centiles for false positives and correct revisits were conservatively increased to one as the centile meant that greater than zero errors were considered impaired. Centiles are rounded to nearest whole number.

**Table 5:** Data for cut offs for impairment per age category (z-scores greater than 1.65 SD from the mean) for each OCS-Plus subtask.

Task	Measure	Aged < 60					Aged > 60 and < 70					Aged > 70				
		*n*	*M*	SD	Min	Max	*n*	*M*	SD	Min	Max	*n*	*M*	SD	Min	Max
Trails	Processing speed	98*	31.44	16.78	16.53	117.92	101	31.67	15.05	15.56	98.34	108	39.54	22.58	14.24	143.38
	Executive score	98*	88.48	18.31	28.57	100	101	77.33	27.90	0	100	108	81.72	25.40	0	100
Rule finding	Accuracy	111	29.17	7.82	3	42	101	26.56	7.99	5	43	108	24.41	8.15	3	41
	Number of rules learned	111	3.34	1.25	0	5	101	2.98	1.30	0	5	108	2.64	1.31	0	5
Figure copy	Figure copy	110	56.85	6.24	19	60	101	55.37	6.09	28	60	104	53.57	7.35	28	60
	Figure recall accuracy	110	47.02	9.70	19	60	101	42.43	11.27	15	60	104	41.25	9.88	13	59
Cancellation	Accuracy	111	29.82	.43	28	30	99	29.63	.62	28	30	107	29.56	.78	27	30
Invisible cancellation	Accuracy	110	28.93	1.43	23	30	100	28.46	1.50	24	30	108	27.94	2.26	13	30

Oxford Cognitive Screen-Plus (OCS-Plus). This table can be used to calculate impairment cut offs. Processing speed is impaired if an individual Z-score is greater than 1.65 SDs from the mean, whereas the remaining tasks are impaired if an individual’s Z-score is lower than − 1.65 SDs from the mean. Asterisks reflect UK only norm data due to unexpected differences between UK and German samples.

Participants were also divided into standard and high education groups, harmonized across the German and UK samples. After correcting for multiple comparisons, sub-task performance was only different between education groups in the Rule Finding task and both versions of the Figure Copy task. It must be noted however, that the normative sample was disproportionately highly educated. This also led to unequal groups, thus not fully allowing for representative splitting into 6 normative groups (i.e., 3 age groups × 2 education level groups). We therefore only present age-related cut-offs in this first instance of normative data and summarise tentative education-based cut offs in Supplementary (see Table S5–S7 and Figure S5).

### Reliability

#### Internal consistency

We used 5000 bootstrapped iterations of split-half reliability analysis to increase robustness of the result. Internal consistency per task was generally good for larger range tasks with most Cronbach alpha values exceeding the standard threshold for good internal consistency (α = 0.70). However, a subset of OCS-Plus tasks was found to have lower alpha values. Specifically, tasks with an inherently low total score variance resulting from a limited number of possible outcome scores (e.g., Picture Naming, Orientation, Semantics, Delayed Recall, and Recognition) were associated with low alpha values. This is likely due to the disproportionate effect of single errors on the consistency score, whereby in low-variance and small-item subtasks a single error will dramatically shift the relative rankings of items^55^. We report the alpha values for each measure for transparency, but, due to test assumptions and variance, we emphasize to only interpret the values which could be computed without error. These are identified in the table with an asterisk. The results of the analyses are presented in Table 6. Note, low variance items were stable across time, discussed next.

**Table 6:** Internal split-half consistency (bootstrapped Cronbach’s alpha) per task in the OCS-Plus from 320 neurologically healthy adults.

Measure	Alpha
Picture naming	0.23
Semantics	0.17
Orientation	0.04
Encoding 1	0.41*
Encoding 2	0.67*
Delayed recall	0.10
Delayed recall and recognition	1*
Incidental episodic memory	0.06
Trails baseline	0.71*
Trails switching	0.87*
Rule finding	1*
Cancellation	0.96*
Invisible cancellation	0.96*
Figure copy	0.94*
Figure Recall	0.93*

Oxford Cognitive Screen-Plus (OCS-Plus). We used 5000 iterations of bootstrapped randomly split-half sampled trail level data to calculate internal consistency. *Refer to reliability ratings we interpret due to the test assumptions being met. Trails baseline and switching conditions are included here to elude to reliability of the executive score which has no trial level data as it is a ratio of baseline and switching performance. Low item sub-tasks have unreliable internal consistency measures due to disproportionate effect of one mistake, making interpretation of the subtask’s reliability difficult, however, test–retest stability suggests all tests are stable across time.

#### Test–retest reliability

A group of 30 healthy ageing controls were retested on the OCS-Plus, on average 320 days apart (SD = 265.89, range = 30–1182), ensuring that they remained neurologically healthy at the second administration by asking about possible neurological events between tests. Test–retest data was collected opportunistically as and when the OCS-Plus was used as part of other projects. Performance in some of the OCS-Plus subtasks was near ceiling in the test–retest cohort. The resultant lack of variance precluded the calculation of correlation or intra-class correlation consistency for these subtasks, though we present correlations corrected for internal consistency, for transparency of the relationships. Consistency at the group level was assessed comparing test–retest performance using a paired sample Wilcoxon test. The subtask test–retest analyses revealed that performance was not statistically different for any of the OCS-Plus tests before and after correction for multiple comparisons (α_corrected_ = 0.003, full results by task given in Supplementary Table S8, including reliable change index data). Therefore, performance on the OCS-Plus subtasks was stable across time.

### Validity

A cohort of 86 German (age: *M* = 68.49, SD = 7.26; higher education 61.63%; 55.81% male) and 73 UK (age: *M* = 61.59, SD = 7.79; higher education 53.49%; 52.23% male) participants completed both the OCS-Plus and the battery of analogous standardized neuropsychological tests. The German cohort completed all of the ACE-R battery, 73 of the UK cohort completed the ACE III Naming, Orientation, Language, and Memory domains. Data from this cohort was used to assess the convergent and divergent validity of the OCS-Plus subtasks. Correlations to assess convergent validity were corrected to account for the internal consistency of each of the tests. Convergent and divergent correlations between subtasks of the OCS-Plus and the validation tests as associated reliability statistics are summarized in Table 7.

**Table 7:** Convergent and divergent correlational analysis (with coefficients correct for internal consistency) of the OCS-Plus sub-tasks.

OCS-Plus subtask	*a*	Task	Convergent validity				Task	Divergent validity			
			*a*	*N*	*r*	*p*		*a*	*N*	*r*	*p*
Picture naming	0.23	ACE Naming	0.80	159	0.30	0.10	BDI	0.86	86	0.12	0.576
		CERAD Boston Naming Task	0.56	86	0.29	0.32	ACE Visuospatial	0.80	159	0.29	0.103
Orientation	0.04	ACE orientation	.80	159	1^a^	.009*	BDI	.86	86	.04	.942
							ACE visuospatial	0.80	159	− 0.01	0.979
Semantics	0.17	ACE language	0.80	159	0.71	< 0.001**	BDI	0.86	86	0.01	0.956
							ACE visuospatial	0.80	159	0.35	0.091
Encoding 1	.41	CERAD 1st Immediate Recall	0.62	86	0.32	0.08	BDI	0.86	86	0.01	0.931
							ACE visuospatial	0.80	159	0.35	0.005
Encoding 2	0.67	CERAD 2nd Immediate Recall	0.62	86	0.38	0.01*	BDI	0.86	86	− 0.1	0.414
							ACE visuospatial	0.80	159	0.24	0.022
Delayed recall	0.10	CERAD Delayed Recall	0.62	86	1^a^	< 0.001**	BDI	0.86	86	− 0.08	0.788
							ACE visuospatial	0.80	159	0.12	0.145
Episodic recognition	0.06	ACE Memory	0.80	159	0.07	0.37	BDI	0.86	86	− 0.63	0.037*
							ACE visuospatial	0.80	159	0.08	0.328
Rule finding	1						BDI	0.86	86	− 0.5	0.135
							ACE language	0.80	159	0.06	0.834
Processing speed	1^+^	CERAD Trail Making Test A	1^+^	86	0.86	0.004*	BDI	0.86	86	0.12	0.136
							ACE memory	0.80	159	− 0.05	0.416
Executive score	1^+^	CERAD Trail Making Test Time Ratio	1^+^	86	0	0.98	BDI	0.86	86	0.05	0.574
							ACE language	0.80	159	− 0.01	0.843
Cancellation	.96	Star Cancellation Missing	.89	85	− 0.24	0.03*	BDI	0.86	85	0.06	0.536
							ACE language	0.80	159	− 0.04	0.63
Invisible cancellation	0.96						BDI	0.86	86	0.05	0.581
							ACE language	0.80	159	− 0.07	0.332
Figure copy accuracy	.94	ROCF Copy	0.60	86	0.32	0.005*	BDI	0.86	86	− 0.02	0.847
							ACE language	0.80	157	0.05	0.510
Figure copy recall	0.93	ROCF 1st Recall	0.80	85	0.20	0.02*	BDI	0.86	86	− 0.15	0.077
							ACE language	0.80	159	− 0.02	0.824

*OCS-Plus* Oxford Cognitive Screen-Plus, *r* Kendall correlation, *BDI* Becks Depression Index, *ACE* Addenbrookes Cognitive Evaluation, *CERAD* Consortium to Establish a Registry for Alzheimer's Disease, *ROCF* Rey–Osterrieth Complex Figure Test. Processing speed is correlated with ACE-Memory as arguably ACE-Visuospatial and -Language are related given anecdotal reports of patients talking themselves through the rule to follow which may add time and visuospatial skills. To check our assumption, we ran correlations between processing speed and both ACE-Visuospatial and -Language and both were small but significant. **Represent alpha corrected significance (convergent = 0.05/13 comparisons, divergent = 0.05/26), *Represents uncorrected alpha level at 0.05. ^+^Refers to assumed internal consistency measure due to measure being unable to generate internal consistency metric, e.g., in the case of time. Correlation coefficients were corrected for internal consistency such that the coefficient = coefficient/square root (internal consistency_x _× internal consistency_y_), where x and y are the OCS-Plus or validation measures. ^a^Refers to corrected correlations that are greater than 1 which suggests the measurement error is not randomly distributed.

Family wise error rate corrections were used to correct for multiple comparisons when evaluating convergent validity and divergent validity. The convergent validation analysis results revealed low, but statistically significant correlations for most tasks pre-alpha correction, and high correlations for other measures including Semantics, Processing speed, Orientation, and Delayed recall. Performance on a few of the OCS-Plus subtasks were not found to be significantly associated with analogous neuropsychological assessments (Table 7), even when taking into account their individual test reliabilities, such as the executive score ratio from the Trail subtask which had a correlation of zero. With regards to divergent validity, we demonstrate no significant correlations with any of the OCS-Plus subtasks, and demonstrate good divergent validity of the OCS-Plus tasks.

### Other potential scoring methods

Lastly, we explored one potential and theoretically motivated methodology for generating cognitive domain cumulative scores which could be used to facilitate data interpretation within clinical settings. Six separate domain-specific scores were generated: executive function, praxis, delayed memory, attention, encoding, and naming and semantic understanding. Measures included in each score, normative data, and corresponding impairment thresholds for these domain total scores are presented in Table 8. This domain scoring system represents one of many potential more global scoring methods. Further research is needed to investigate the utility of the proposed alternative scoring methods, particularly with regards to specific clinical populations.

**Table 8:** Data of scores and cut offs for impairment (scores lower than 5th centile) for OCS-Plus cognitive domains.

Summative score	Included measures	N	Med	Min	Max	Cent.	*M*	SD
Executive function	Rule finding	319	121.71	19	148	122	111.31	29.14
	Rules learned							
	Trails exec. score							
	False positives							
Praxis	Figure copy	314	103	39	120	103	99.09	15.34
	Figure recall							
Delayed memory	Delayed recall	320	9	3	10	9	9.02	1.32
	Delayed recall and recognition							
Attention	Cancellation	316	59	40	60	59	58.13	2.1
	Invisible cancellation							
Memory encoding	Encoding 1	320	10	4	10	10	9.24	1.11
	Encoding 2							
Picture naming and semantic understanding	Picture naming	320	8	4	8	8	7.78	.54
	Semantics							

Oxford Cognitive Screen-Plus (OCS-Plus). Domains were theoretically grouped by OCS-Plus sub-task design into cognitive domains, which were used to generate domain scores for per participant. Code and exact calculations are found in our data scripts (https://osf.io/cfmwk/?view_only=7e5cdf8d963b46568a5069b50c3b4e76). *Cent* refers to 5th centile (1.65 SDs from mean).

## Discussion

We presented normative data for a novel, tablet-based brief cognitive assessment aiming to sensitively detect fine-grained impairments within ageing adults. Age group specific cutoffs were established for each of the OCS-Plus subtasks, based on data from a cohort of neurologically healthy older adults. The validity of the OCS-Plus subtasks was then evaluated against a series of analogous standard neuropsychological assessments. The OCS-Plus subtasks were found to have good divergent validity. Performance on many OCS-Plus subtasks was found to correlate with performance on analogous standard measures, though some of the convergent validity in this healthy ageing cohort was relatively low. The OCS-Plus was found to have good test–retest reliability. The present paper and data present the first step towards building clinically valid tools and further research is underway on the more easily distributable Android app to expand the normative data and allow both age and education specific norms. Importantly, further research into OCS-Plus validation in clinical groups is required.

### Normative data

The UK and German cohort of healthy ageing adults included in this investigation were collectively found to perform well on the OCS-Plus subtasks. The lack of floor effects and significant variance present within the normative scores for most subtasks are promising signs of a sensitive test. Equally, OCS-Plus includes more straightforward tasks like Picture Naming, Orientation and Episodic Recognition, where healthy participants' performance was found to reach ceiling with a comparatively small range of potential total score outcomes. These tasks are included to allow assessment across a range of abilities, and these are more likely to be of interest in screening for a more apparent cognitive impairment. When these scores are considered in the broader context of OCS-Plus performance, they may allow excluding a more severe impairment diagnosis or identifying larger changes in cognitive abilities over time. For example, they might be useful for differentiation of patients with slight and specific vs. more severe and global deficits.

Performance on OCS-Plus subtasks was found to be significantly different between various age groups, and normative cut offs for ages < 60, 60–70 and > 70 are provided. The grouping according to age happened post-hoc to split the data across age groups of comparable sizes and this initial normative data did not span the entire education spectrum. Our sample was unequally distributed for full age-and education combined cut offs. And though only small differences between the two education levels appeared present at this time, visualization of the data as well as findings of age and education associations with OCS-Plus tasks in a large cohort in rural South Africa spanning the full spectrum of education^18^, suggests further data is needed here. Performance on the OCS-Plus subtasks with a restricted range of outcome scores (e.g., Picture Naming) was not found to differ significantly between age and education groups. This finding agrees with the conceptualization of these specific subtasks as qualitative rather than quantitative metrics, with neurologically healthy adults performing at ceiling here. Future studies are invited to continuously extend the norm data which will enable us to divide participants in more narrow age and education groups and define their cut-off values in a dynamically evolving normative base. Automatic reporting within the Android App will allow even closer matching of each participant to their relevant normative control group.

### Reliability

The OCS-Plus subtasks were found to demonstrate good test–retest reliability at the group level, despite the wide range of test–retest intervals. Values for some subtasks were low due to inherent low variance. However, performance on OCS-Plus subtasks, overall, was found to be stable across time within this investigation’s neurologically healthy ageing participant sample. Internal consistency per task was generally good for tasks of larger range (e.g., not Picture Naming, Orientation, Semantics etc.) with most Cronbach alpha values exceeding 0.70). It is worth noting that these simple tasks are included as basic checks whether participants and patients are able to name pictures of stimuli, select pictures based on presented words, and orient themselves. This is to establish a baseline performance of core general abilities to then more sensitively assess executive functioning and memory. In addition, starting the testing session with these subtasks ensures a low barrier of entry and makes participants feel comfortable with the testing situation and interacting with the tablet.

However, reliability statistics could not be validly calculated for subtasks with restricted possible total scores as participants' scores were at ceiling. Collectively, the reliability analyses conducted in this study suggest that the OCS-Plus represents a reliable neuropsychological assessment battery.

### Validity

The convergent and divergent validity of the OCS-Plus subtasks was evaluated by comparing performance on these tasks to performance on analogous, standardized neuropsychological measures, and correcting the correlation coefficient for the reliability of the tests/subtasks. The majority of these comparisons had comparatively low (< 0.50) correlation coefficients, possible due to low variance in ceiling type performance of the control participants. However, in terms of size of the convergent correlations most were at or above an acceptable level of convergence seen in validations of other widely used similar screens used in this investigation, i.e., > 0.20 (e.g.^46^). This suggests that, like other screen tasks, while performance on the OCS-Plus subtasks and analogous neuropsychological metrics is significantly associated, these tests are not exactly identical or had too few lower range scores to compute reliable estimates (e.g., Picture Naming, Orientation, Semantics etc.).

Some difference in performance between OCS-Plus and pen-and-paper tasks is expected, as the stimuli, experimental design, and difficulty level are similar, but not identical across these assessments. Further research in clinical groups, with larger variance across both OCS-Plus and standardized convergent validity tasks is called for. As a whole, OCS-Plus subtasks were found to have good divergent validity versus assessments aiming to test theoretically unrelated constructs.

### Potential summative scores and clinical application

The OCS-Plus outputs a detailed, task-specific performance summary following the completion of each patient assessment in a brief overview snapshot (see Fig. 1). We have also suggested one potential method for combining test scores across cognitive domains and have provided normative data cut-offs for using this alternate scoring approach. This method is described as one of many potential alternative clinician-focused OCS-Plus scoring systems. Future research is needed to investigate the utility of any domain scoring system, particularly in relation to specific clinical groups and to identify other informative alternate scoring methods.

### Study limitations and future research

The OCS-Plus is not meant to provide a method for separating the spectrum of cognitive decline into arbitrary impairment classification groups. Instead, it is designed as a tool for briefly measuring more detailed cognitive performance metrics for individual patients, which can then be employed to inform clinical decision making. The boundaries distinguishing normal, age-related cognitive decline from abnormal cognitive deficits are not clearly established and the OCS-Plus in its current state is not an appropriate tool for allocating patients to specific clinical groups. Further research is required into OCS-Plus validation for cohorts diagnosed with specific pathologies.

The OCS-Plus outputs a wide range of performance metrics, a subset of which were introduced and evaluated in the present paper. Most OCS-Plus subtasks record detailed information including the x, y coordinates and timestamps of each participant response as well as audio recordings of each task (recordings start when a subtask is begun and end when a subtask is finished). These more complex performance metrics can be analyzed to provide a more detailed analysis of participant performance. For example, spatial search strategy could be quantified based on responses within the selection and figure copy task and this data could be analyzed to evaluate task planning and organizational abilities. Additional research is needed to explore these potentially informative extensions of OCS-Plus functionality.

It must be noted that we developed the OCS-Plus using the Matlab application described in this paper. Future releases will be available on the minimally different Android app (i.e., no change in instructions or task stimuli).

Further, four characteristics our sample potentially hamper generalizing the results on a population level. First, our sample was highly educated, as such this restricts confident interpretation of an individual’s performance where they have low levels of education. Indeed, we have previously found very clear age and education effects in the rural South African cohort^18^, demonstrating the sensitivity of the measures and making explicit the need for age and education specific cut-offs, especially where the range of education levels include such extremes as ‘no formal education’^18^. However, we note that the cohort used in the validation were more evenly distributed between standard and higher education. Second, our sample does not include people from different ethnicities. Third, our sample as a whole was not pre-screened for cognitive impairments and where it was, it was done so with a crude cognitive screen. Experimenters relied on self-reports regarding previous neurological and psychiatric problems. It is possible that some of these individuals were characterized by subtle cognitive changes and/or that some participants may have lacked insight into these changes. As a whole, performance on the validation tasks did not indicate any gross impairment. Lastly, test–retest reliability was assessed based on opportunity data which led to a wider range of inter-test intervals. Whilst the present data provide first insights into the reliability of the OCS-Plus over time, future studies are needed to assess test–retest reliability in standardized and clinically relevant intervals. We hope that any potential small sources of noise in the normative data will even out as even larger normative samples will be collected. Future research should include samples with a wider variety of ethnicity and education levels to ensure appropriately matched test cut-offs are available for use across the full population.

### The road ahead

OCS-Plus will be made available as an Android app to be downloaded on various tablet types. We anticipate that updated versions will include even larger age-education normative comparison groups as data collection is ongoing and the Android app is already set up for these updates as it facilitates anonymized data sharing. All the current data has been made openly available on the Open Science Framework, and we intend to update this data in a transparent and open way.

Similar to the English, Shangaan, and German versions, the app has been set up to allow different language and cultural adaptations to be made. Several further translations of OCS-Plus are in the making, each with respective normative data.

Finally, given the increasing need for remote assessment, developments on adapting OCS-Plus for remote assessments are planned.

## Conclusion

The present study presented a first set of healthy ageing normative data for the OCS-Plus, demonstrating test reliability and initial validity of this novel, brief tablet-based cognitive assessment in a neurologically healthy ageing cohort. This assessment tool can be used to create informative summaries of finer-grained cognitive impairments in healthy ageing and clinical groups. Future research should aim to establish the feasibility of the OCS-Plus in various clinical cohorts.

## Supplementary Information

Supplementary Information.

## Data Availability

The data, codebook, and analysis scripts of this project are publicly available on the Open Science Framework (OSF) (https://osf.io/cfmwk/) with the DOI https://doi.org/10.17605/OSF.IO/CFMWK.
