# Supplementary material for: Introducing the tablet-based Oxford Cognitive Screen-Plus (OCS-Plus) as an assessment tool for subtle cognitive impairments
Source: Sci Rep. 2021 Apr 12;11:8000. doi: 10.1038/s41598-021-87287-8 (PMC8041764; doi:10.1038/s41598-021-87287-8)
Supplement: Supplementary file 1 — Supplementary Information. [file 41598_2021_87287_MOESM1_ESM.docx]

Introducing the tablet-based Oxford Cognitive Screen-Plus (OCS-Plus) as an assessment tool for subtle cognitive impairments.

*Nele Demeyere^1^, Marleen Haupt^2^, Sam Webb^1^, Lea Strobel^2^, Elise Milosevich^1^, Margaret J Moore^1^, Hayley Wright^3^, Kathrin Finke^4*^, Mihaela D Duta^1*^*

*^1^ Department of Experimental Psychology, University of Oxford*

*^2^ Department of General and Experimental Psychology, Ludwig-Maximilians-Universität München, Munich, Germany*

*^3^ Coventry University*

*^4^ Hans-Berger Department of Neurology, University Hospital Jena, Jena, Germany*

* shared senior authorship

Correspondence to:

Nele Demeyere

Department of Experimental Psychology

New Radcliffe House, Radcliffe Observatory Quarter

Oxford, OX2 6AE

Tel: 01865 271340

Email: [nele.demeyere@psy.ox.ac.uk](mailto:nele.demeyere@psy.ox.ac.uk)

**Age and Education distributions**


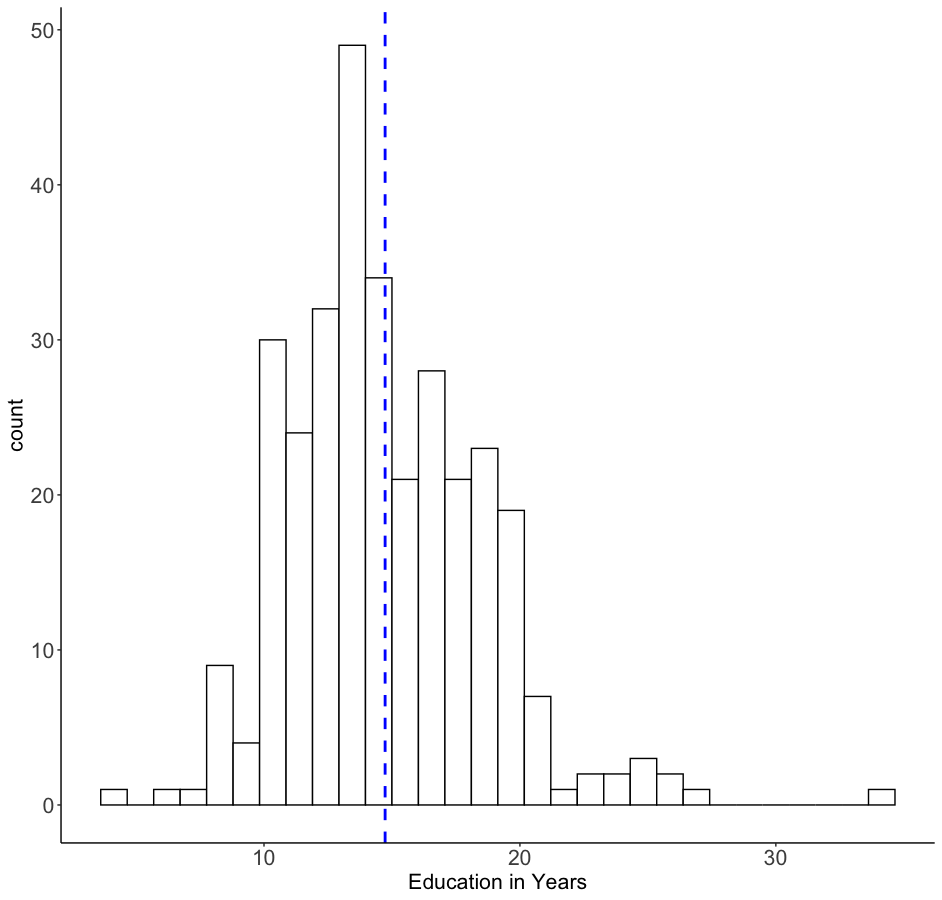

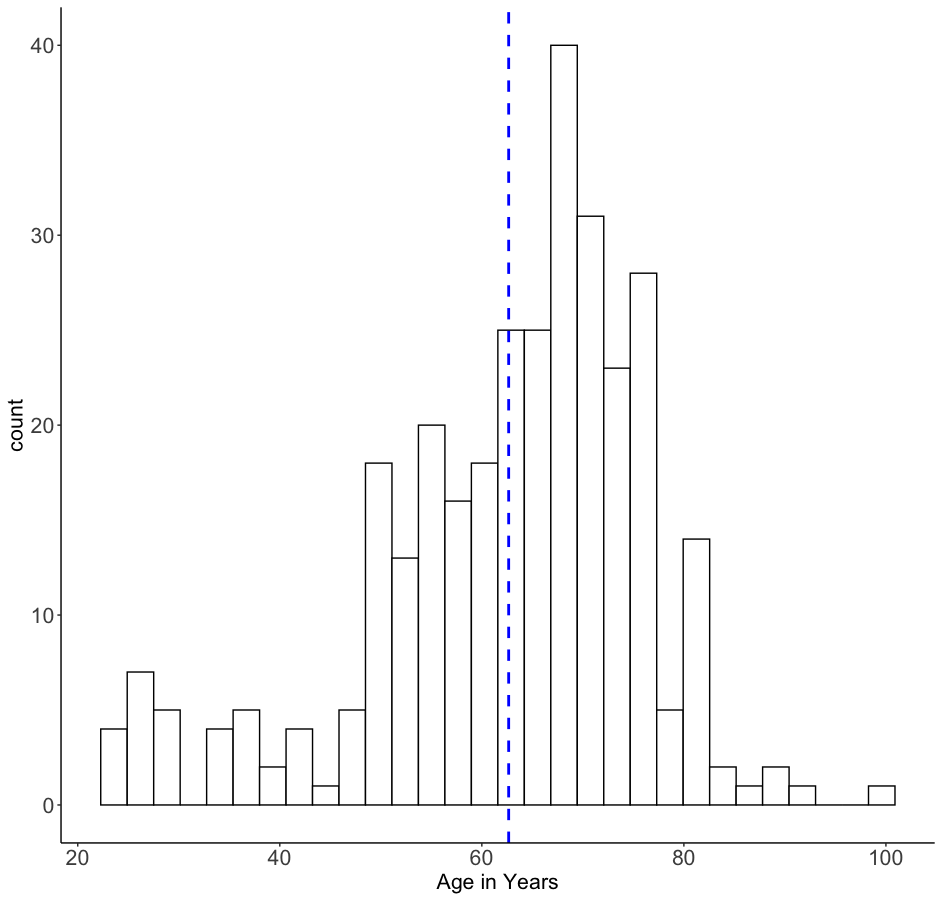


*Figure S1*. Illustrates the underlying distributions of years of education and years of age in the large neurologically healthy control sample (*N*=320). Figure created in RStudio (version 3.5.1^47^) using ggplot2^50^ (version 3.3.2)**,** figure available at <https://osf.io/j6t2u/> under a CC-BY4.0 license.

**German and UK sample differences**

Table S1

*Wilcoxon rank sum test with continuity correction comparisons between German (n=* 86) *and UK (n=234) cohorts on each OCS-Plus subtask*

|  | German | | UK | |  |  |
| --- | --- | --- | --- | --- | --- | --- |
| Measure | *M* | *SD* | *M* | *SD* | *p* | *r* |
| Picture Naming | 3.85 | .36 | 3.91 | .34 | .036* | .04* |
| Semantics | 3.93 | .30 | 3.87 | .36 | .096 | .10 |
| Orientation | 3.98 | .15 | 3.88 | .33 | .014* | .01* |
| Encoding 1 | 4.51 | .68 | 4.30 | .88 | .103 | .10 |
| Encoding 2 | 4.90 | .31 | 4.87 | .50 | .659 | .66 |
| Episodic Recognition | 3.50 | .63 | 3.59 | .56 | .309 | .31 |
| Delayed Recall | 4.03 | 1.14 | 4.12 | 1.20 | .281 | .28 |
| Delayed Recall Recognition | 4.94 | .24 | 4.91 | .31 | .357 | .36 |
| Rule Finding | 25.63 | 7.24 | 27.15 | 8.51 | .084 | .08 |
| Rules Learned | 2.77 | 1.18 | 3.07 | 1.36 | .022* | .02* |
| Trails Exec Score | 75.39 | 27.75 | 84 | 23.61 | .003* | <.001 |
| Trails Processing Speed | 30.99 | 16.90 | 34.87 | 19.25 | .008* | .01* |
| Cancellation | 29.65 | .57 | 29.68 | .65 | .264 | .26 |
| Cancellation False Positives | .07 | .26 | .08 | .27 | .823 | .82 |
| Invisible Cancellation | 28.29 | 1.53 | 28.50 | 1.91 | .053 | .05 |
| Invisible Cancellation Revisits | .59 | 1.30 | .39 | 1.13 | .003* | <.001 |
| Figure Copy | 54.16 | 6.67 | 55.72 | 6.67 | <.001** | <.001 |
| Figure Recall | 42.56 | 9.58 | 44.05 | 10.90 | .110 | .11 |

*Note*. Oxford Cognitive Screen – Plus (OCS-Plus). Significance at the alpha corrected level (.05/18) are denoted with **, significance at the .05 level are represented by a single *. Pearsons *r* is used for effect size.

Table S2

*Wilcoxon rank sum test with continuity correction for German (n=* 86) *and UK (n=234) cohorts on each OCS-Plus subtask divided into three age groups*

|  | <60  *n*=111 | | | | | 60-70  *n*=101 | | | | | >70  *n*=108 | | | | |
| --- | --- | --- | --- | --- | --- | --- | --- | --- | --- | --- | --- | --- | --- | --- | --- |
|  | mean | | cut-off | |  | mean | | cut-off | |  | mean | | cut-off | |  |
| Task | UK | Ger | UK | Ger | *p* | UK | Ger | UK | Ger | *p* | UK | Ger | UK | Ger | *p* |
| Picture Naming | 3.91 | 3.92 | <3 | <4 | .959 | 3.93 | 3.87 | <3 | <3 | .355 | 3.91 | 3.81 | <3 | <3 | .084 |
| Orientation | 3.94 | 4 | <3 | <4 | .367 | 3.89 | 4 | <3 | <4 | .052 | 3.80 | 3.95 | <3 | <4 | .044* |
| Semantics | 3.89 | 3.85 | <3 | <3 | .779 | 3.87 | 3.90 | <3 | <3 | .785 | 3.83 | 3.98 | <3 | <4 | .033* |
| Encoding 1 | 4.53 | 4.62 | <3 | <4 | .735 | 4.29 | 4.55 | <3 | <4 | .225 | 3.98 | 4.45 | <2 | <3 | .016* |
| Encoding 2 | 4.98 | 4.92 | <5 | <5 | .246 | 4.83 | 4.90 | <4 | <4 | .917 | 4.76 | 4.88 | <3 | <4 | .336 |
| Delayed Recall | 4.45 | 4.38 | <3 | <3 | .747 | 4.01 | 4.13 | <2 | <3 | .680 | 3.76 | 3.86 | <=1 | <1 | .939 |
| Delayed Recall Recognition | 4.93 | 5 | <4 | <5 | .327 | 4.93 | 4.94 | <4 | <5 | .907 | 4.85 | 4.93 | <4 | <4 | .292 |
| Episodic Recognition | 3.57 | 3.46 | <3 | <3 | .567 | 3.64 | 3.45 | <3 | <3 | .126 | 3.56 | 3.55 | <3 | <2 | .852 |
| Trails Processing Speed | 31.44 | 21.09 | >19.26 | 12.84 | .001** | 32.83 | 29.05 | >20.04 | >15.97 | .065 | 42.13 | 35.48 | >20.81 | >17.64 | .069 |
| Trails Exec Score | 88.48 | 63.96 | <49.42 | 23.33 | .001** | 79.71 | 71.95 | <25 | <17.42 | .131 | 81.88 | 81.46 | >32.14 | <33.81 | .650 |
| Cancellation | 29.85 | 29.62 | <29 | 28.60 | .116 | 29.67 | 29.53 | <28 | <29 | .214 | 29.45 | 29.74 | <27 | <29 | .159 |
| Cancellation False positives | 0 | 0 | >1 | >1 | .313 | 0 | 0 | >1 | >1 | .489 | 0 | 0 | >1 | <=1 | .115 |
| Invisible Cancellation | 29 | 28.38 | <27 | <26 | .063 | 28.51 | 28.35 | <25 | <26 | .504 | 27.76 | 28.21 | <24 | <26 | .551 |
| Invisible Cancellation Revisits | 0 | 0 | >1 | >2 | .004* | 0 | 0 | >3 | >2 | .061 | 1 | 1 | >4 | >3 | .771 |
| Figure Copy | 56.75 | 57.62 | <41 | <54 | .143 | 55.54 | 54.97 | <41 | <48 | .044* | 54.29 | 52.50 | <39 | <33 | .070 |
| Figure Recall | 47.02 | 47 | <27 | <38 | .569 | 42.34 | 42.61 | <17 | <23 | .699 | 41.32 | 41.14 | <20 | <17 | .858 |

*Note*. Oxford Cognitive Screen - Plus (OCS-Plus). *Centile* refers to either 5^th^ or 95^th^ centile for classifying impairment, where all measures are 5^th^ centile or below for cut off except for the following tasks which are 95^th^ centile or above: trails processing speed, cancellation false positives, and invisible cancellation revisits. Centiles per age group are divided into UK and German sample generated centile cut offs (not inclusive of value). *p* refers to significance on Wilcoxon rank sum test with continuity correction between means of the two samples within each age group. We report means on each task per country specific samples per task separated into age groups. We have separate centiles to show that in cases where there are significant differences the centile cut off is or is not different between samples. Significance at the alpha corrected level (.05/18) are denoted with **, significance at below .05 is *. Values were rounded to nearest whole number if the value was not an available score on a subtask.

Table S3

*Wilcoxon rank sum test with continuity correction for German (n=* 86) *and UK (n=234) cohorts on each OCS-Plus subtask divided into standard and high education groups*

|  | Standard Education (*n*=84) | | | | | High Education (*n*=232) | | | | |
| --- | --- | --- | --- | --- | --- | --- | --- | --- | --- | --- |
|  | mean | | centile | |  | mean | | centile | |  |
|  | UK | German | UK | German | *p* | UK | German | UK | German | *p* |
| Picture Naming | 3.92 | 3.85 | <3 | <3 | .297 | 3.91 | 3.85 | <3 | <3 | .09 |
| Orientation | 3.80 | 3.97 | <3 | <4 | .045 | 3.91 | 3.98 | <3 | <4 | .07 |
| Semantics | 3.80 | 3.94 | <3 | <4 | .086 | 3.89 | 3.92 | <3 | <4 | .34 |
| Encoding 1 | 3.98 | 4.45 | <2 | <3 | .044 | 4.40 | 4.55 | <3 | <3 | .37 |
| Encoding 2 | 4.84 | 4.85 | <4 | <4 | .546 | 4.88 | 4.92 | <4 | <4 | .92 |
| Delayed Recall | 3.80 | 3.76 | <=1 | <=1 | .666 | 4.22 | 4.21 | <2 | <3 | .63 |
| Delayed Recall Recognition | 4.86 | 4.91 | <4 | <4 | .530 | 4.92 | 4.96 | <4 | <5 | .31 |
| Episodic Recognition | 3.51 | 3.33 | <3 | <2 | .216 | 3.61 | 3.60 | <3 | <3 | .87 |
| Trails Processing Speed | 33.10 | 36.08 | >18.49 | 17.05 | .721 | 35.35 | 27.82 | 20 | 15.04 | 0 |
| Trails Exec Score | 81.32 | 68.53 | <21.98 | 11.90 | .077 | 85 | 79.65 | 28.57 | 34.76 | .03 |
| Cancellation | 29.65 | 29.67 | <28 | <29 | .814 | 29.69 | 29.63 | <28 | <29 | .25 |
| Cancellation False positives | 0 | 0 | >1 | >0 | .163 | 0 | 0 | >1 | >1 | .409 |
| Invisible Cancellation | 28.33 | 28.30 | <25 | <26 | .703 | 28.56 | 28.28 | <25 | <26 | .047 |
| Invisible Cancellation Revisits | 0 | 1 | >3 | >3 | .059 | 0 | 0 | >3 | >2 | .046 |
| Figure Copy | 53.31 | 54.73 | <37 | <45 | .902 | 56.31 | 53.81 | <42 | <33 | .000 |
| Figure Recall | 37.62 | 41.18 | <17 | <18 | .200 | 45.85 | 43.42 | <27 | <26 | .045 |

*Note*. Education is split into those who completed further education or those who only completed the legal standard of education for their age in each country. Oxford Cognitive Screen - Plus (OCS-Plus). *Centile* refers to either 5^th^ or 95^th^ centile for classifying impairment, where all measures are 5^th^ centile or below for cut off except for the following tasks which are 95^th^ centile or above: trails processing speed, cancellation false positives, and invisible cancellation revisits. Centiles per age group are divided into UK and German sample generated centile cut offs (not inclusive of value). *p* refers to significance on Wilcoxon rank sum test with continuity correction between means of the two samples within each education group. We report means on each task per country specific samples per task separated into education groups. We have separate centiles to show that in cases where there are significant differences the centile cut off is or is not different between samples. Significance at the alpha corrected level (.05/18) are denoted with **, significance at below .05 is *.

**Convergent and Divergent Correlation Plots**

Here we present the overview of measures for convergent and divergent validation included in the Oxford Cognitive Screen - Plus (OCS-Plus) paper. Further we present relevant plots of validation analysis between convergent and divergent tasks against OCS-Plus sub measures.


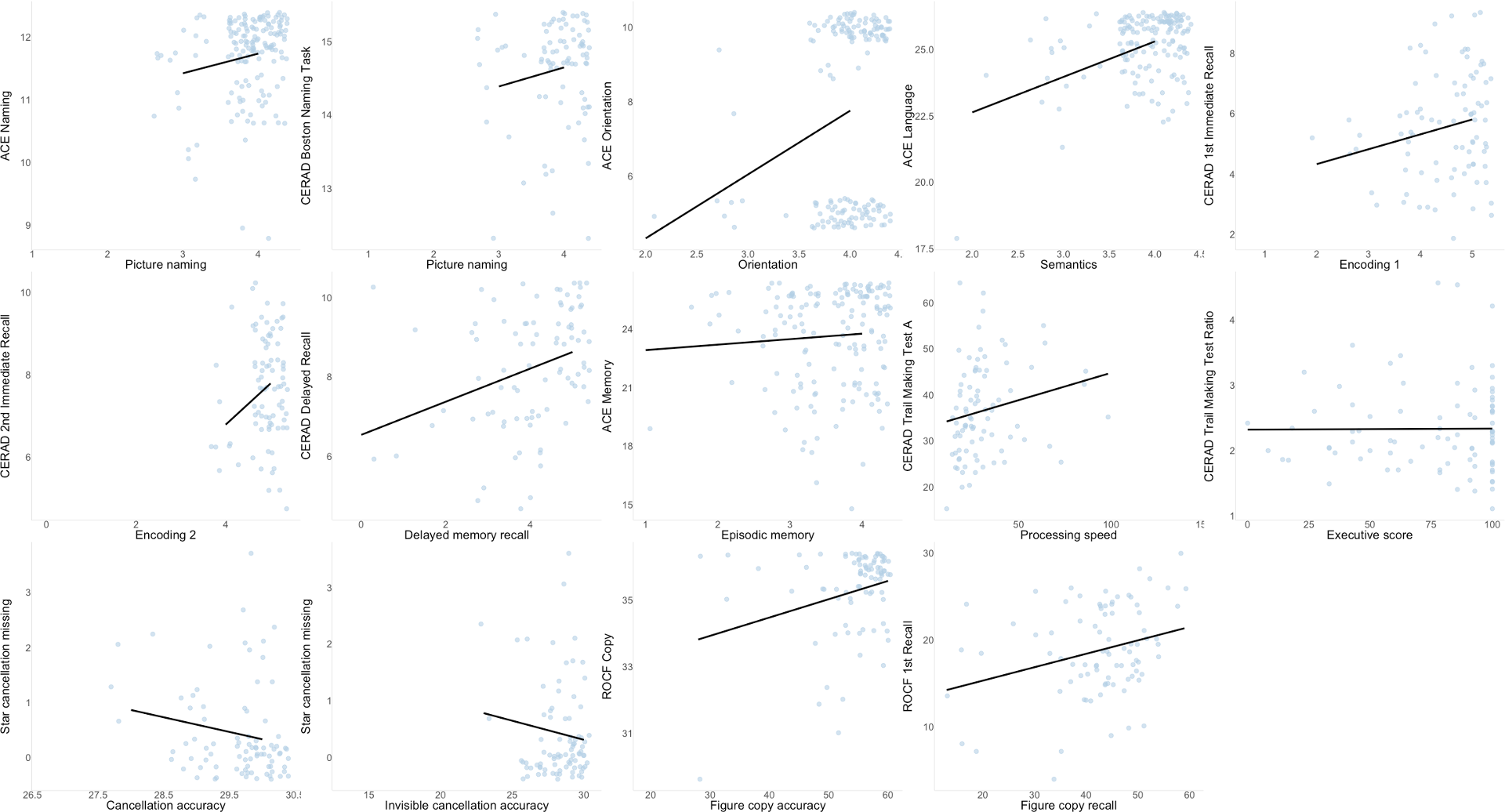
*Figure S2*. Convergent validity scatter plots between OCS-Plus measures and comparator measures, line representation linear model of best fit. To aid visualisation, jitter is imposed to widen and reveal overlapping figures, this means not all values represent real values, but the plots do maintain real patterns of data. Figure created in RStudio (version 3.5.1^47^) using ggplot2^50^ (version 3.3.2)**,** figure available at <https://osf.io/fxg3k/> under a CC-BY4.0 license.


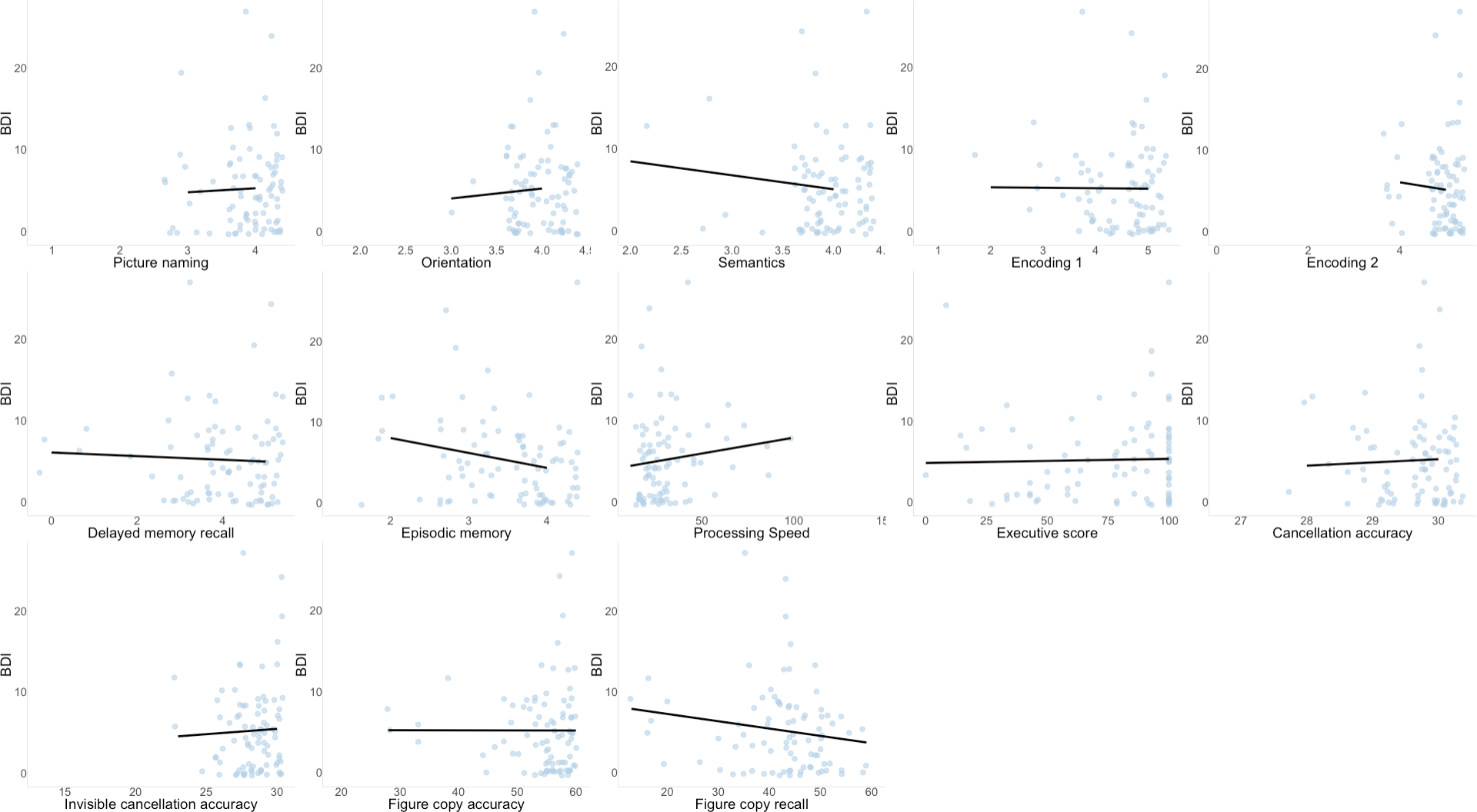
*Figure S3.*  Divergent validity scatter plots between OCS-Plus measures and Becks Depression Inventory (BDI). Line represents linear model of best fit. To aid visualisation, jitter is imposed to widen and reveal overlapping figures, this means not all values represent real values, but the plots do maintain real patterns of data. Figure created in RStudio (version 3.5.1^47^) using ggplot2^50^ (version 3.3.2)**,** figure available at <https://osf.io/q8sc3/> under a CC-BY4.0 license.


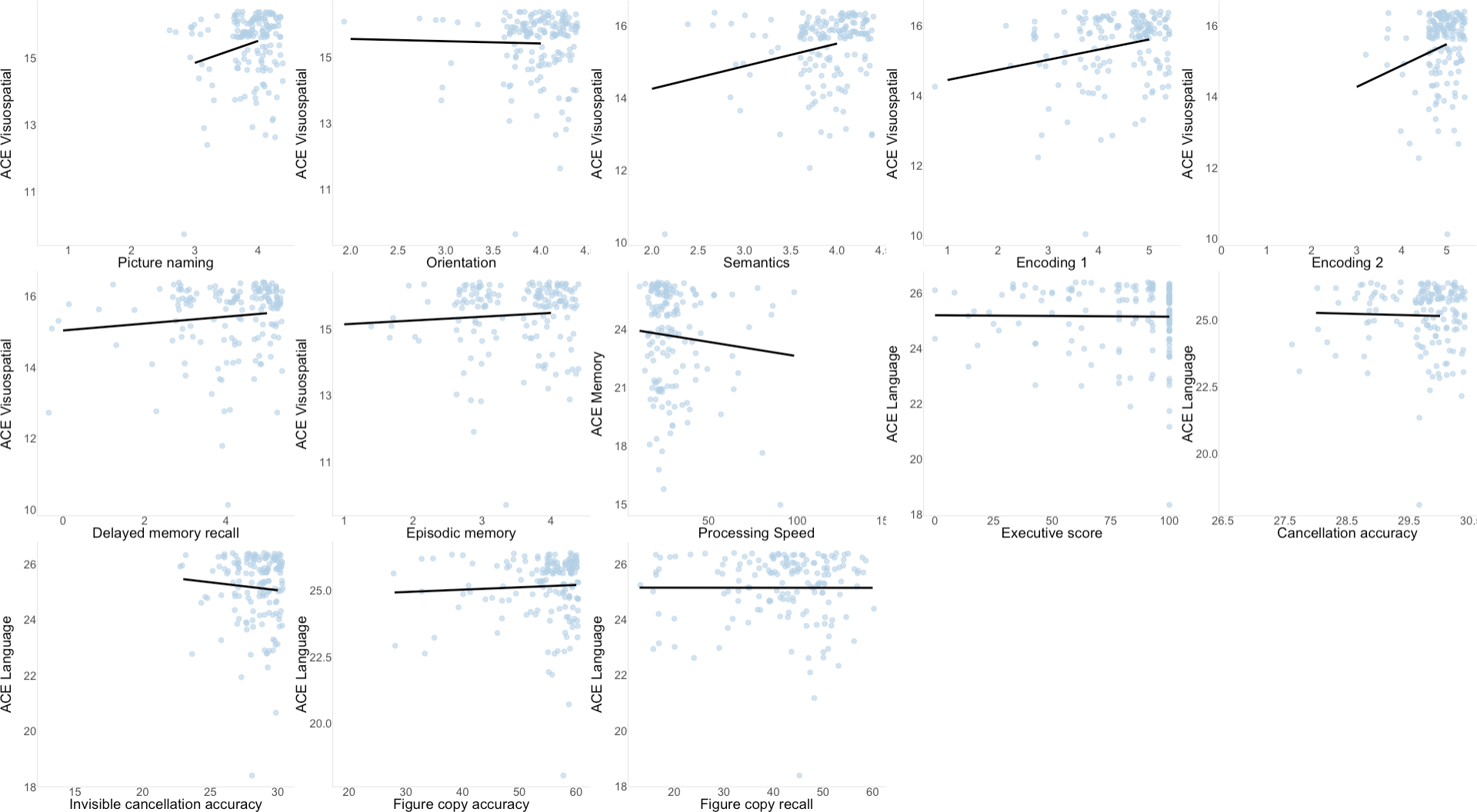
*Figure S4.*  Divergent validity scatter plots between OCS-Plus measures and Addenbrookes Cognitive Examination summative scores. Line represents linear model of best fit. To aid visualisation, jitter is imposed to widen and reveal overlapping figures, this means not all values represent real values, but the plots do maintain real patterns of data. This is 2/2 part of the divergent validity graphs. Figure created in RStudio (version 3.5.1^47^) using ggplot2^50^ (version 3.3.2)**,** figure available at <https://osf.io/q5auy/> under a CC-BY4.0 license.

Table S4

*Overview of measures compared for convergent and divergent validation analyses against OCS-Plus tasks with range values*

| OCS-Plus subtask | Min | Max | Convergence task | Min | Max | Divergence task | Min | Max |
| --- | --- | --- | --- | --- | --- | --- | --- | --- |
| Picture Naming | 0 | 4 | ACE Naming | 0 | 12 | BDI | 0 | 63 |
|  |  |  | CERAD Boston Naming Task | 0 | 27 | ACE Visuospatial | 0 | 16 |
| Orientation | 0 | 4 | ACE Orientation | 0 | 26 | BDI |  |  |
|  |  |  |  |  |  | ACE Visuospatial |  |  |
| Semantics |  |  | ACE Language | 0 | 26 | BDI |  |  |
|  | 0 | 5 | ACE Fluency | 0 | 5 | ACE Visuospatial |  |  |
| Encoding 1 |  |  | CERAD 1st Immediate Recall | 0 | 9 | BDI |  |  |
|  |  |  |  |  |  | ACE Visuospatial |  |  |
| Encoding 2 | 0 | 5 | CERAD 2nd Immediate Recall | 0 | 10 | BDI |  |  |
|  |  |  |  |  |  | ACE Visuospatial |  |  |
| Delayed Recall | 0 | 5 | CERAD Delayed Recall | 0 | 10 | BDI |  |  |
|  |  |  |  |  |  | ACE Visuospatial |  |  |
| Episodic Recognition | 0 | 4 | ACE Memory | 0 | 26 | BDI |  |  |
|  |  |  |  |  |  | ACE Visuospatial |  |  |
| Processing speed | 0 | inf | CERAD Trail Making Test A | 0 | inf | BDI |  |  |
| Executive score | 0 | 100 | CERAD Trail Making Test Time Ratio | 0 | inf | ACE Language | 0 | 26 |
| Cancellation | 0 | 30 | Star Cancellation Missing | 0 | 56 | BDI |  |  |
|  |  |  |  |  |  | ACE Language |  |  |
| Invisible Cancellation | 0 | 30 | Star Cancellation Missing | 0 | 56 | BDI |  |  |
|  |  |  |  |  |  | ACE Language |  |  |
| Figure copy accuracy | 0 | 60 | ROCF Copy | 0 | 36 | BDI |  |  |
|  |  |  |  |  |  | ACE Language |  |  |
| Figure copy recall | 0 | 60 | ROCF 1st Recall | 0 | 30 | BDI |  |  |
|  |  |  |  |  |  | ACE Language |  |  |

*Note*. Oxford Cognitive Screen - Plus (OCS-Plus). ACE refers to Addenbrookes Cognitive Examination battery. CERAD refers to Consortium to Establish a Registry for Alzheimer's Disease. ROCF refers to Rey–Osterrieth Complex Figure Test. Each suffix to ‘ACE’ refers to subsection of ACE battery. Min and Max values are not repeated for repeated validation tests. Min and max values are true to task and not from observed data. Convergence task refers to comparisons where we expect a significant positive correlation and divergence task refers to where we expect no significant relationship between tasks. *Inf* refers to infinite limit on values.

**Age and Education Differences**

Comparisons between age and education groups on each measure. Double asterisk in any table represents significance at the family-wise corrected alpha level (alpha correct per number of tasks compared within an analysis), whereas single asterisk reflections uncorrected alpha level significance below .05. Further we present data for the education groups in Table S3. Finally, we present age and education grouped performance on the subtasks visually in Figure S1.

Table S5

*Age category differences on each OCS-Plus subtask using a linear model within a combined sample of neurologically healthy adults (N=320)*

| Task | *F* value | *p* value | Eta |
| --- | --- | --- | --- |
| Picture Naming | .48 | .616 | 0 |
| Orientation | 2.34 | .098 | .01 |
| Semantics | .01 | .986 | 0 |
| Encoding 1 | 5.62 | .004* | .03 |
| Encoding 2 | 4.02 | .019* | .02 |
| Delayed Recall | 8.71 | <.001** | .05 |
| Delayed Recall and Recognition | 1.28 | .281 | .01 |
| Episodic Recognition | .09 | .917 | 0 |
| Processing Speed | 8.12 | <.001** | .05 |
| Trails Executive Score | 2.93 | .055 | .02 |
| Rule Finding | 9.78 | <.001** | .06 |
| Rules Learned | 8.14 | <.001** | .05 |
| Figure Copy | 6.68 | .001** | .04 |
| Figure Recall | 9.44 | <.001** | .06 |
| Cancellation | 5.1 | .007* | .03 |
| False Positives | .05 | .954 | 0 |
| Invisible Cancellation | 8.52 | <.001** | .05 |
| Correct Revisits | 10.11 | <.001** | .06 |

*Note*. Oxford Cognitive Screen - Plus (OCS-Plus). Age categories are <60 (*n*=111), 60-70(*n*=101), and >70 (*n*=108). One asterisk refers to significance at the conventional .05 level, two asterisks refer to significance at the alpha correct level of .05/18 comparisons on a one-way ANOVA test. To see mean and standard deviation values for each age group per task, see Table S2.

Table S6

*Education category differences on each OCS-Plus sub-measure Wilcoxon rank sum test with continuity correction*

| Task | *U* value | *p* value | *r* |
| --- | --- | --- | --- |
| Picture Naming | 9591 | .68 | -.02 |
| Orientation | 9331 | .24 | -.07 |
| Semantics | 9252 | .20 | -.07 |
| Encoding 1 | 8375 | .03* | -.12 |
| Encoding 2 | 9334.5 | .25 | -.06 |
| Delayed Recall | 789.5 | .01* | -.16 |
| Delayed Recall and Recognition | 9261 | .16 | -.08 |
| Episodic Recognition | 8279 | .02* | -.13 |
| Processing Speed | 9641.5 | .89 | -.01 |
| Trails Executive Score | 823.5 | .03* | -.12 |
| Rule Finding | 6695.5 | <.001** | -.24 |
| Rules Learned | 7026 | <.001** | -.22 |
| Figure Copy | 7077 | <.001** | -.19 |
| Figure Recall | 6567.5 | <.001** | -.23 |
| Cancellation | 9688.5 | .98 | 0 |
| False Positives | 9884 | .67 | .02 |
| Invisible Cancellation | 8961.5 | .28 | -.06 |
| Correct Revisits | 10504 | .12 | .09 |

*Note*. Oxford Cognitive Screen - Plus (OCS-Plus). Education split into country specific standard education (*n*=84) and higher education (*n*=232). One asterisk refers to significance at the conventional .05 level, two asterisks refer to significance at the alpha correct level of .05/18 comparisons on a Wilcoxon rank sum test with continuity correction. Within the UK cohort, participants with 12 or fewer years of education were considered to be within the standard education group and participants with more than 12 years were allocated to the high education group. These cutoffs are based on the UK legal education requirements, which stipulate that individuals must remain in school for at least 12 years. Due to different regulations between countries, German participants were binarised as standard education if they did not complete higher education and classed as highly educated if they completed further education. To see mean and standard deviation values for each age group per task, see Table S3.

Table S7

*Normative Data and Cut Offs for Impairment per Education category (Z-scores Greater Than 2SD From the Mean or Scores Lower than 5^th^ centile) from a combined sample of UK and German neurologically healthy adults (N=320)*

|  |  | Standard education (*n*=84) | | | | | | | High education (*n*=232) | | | | | | |
| --- | --- | --- | --- | --- | --- | --- | --- | --- | --- | --- | --- | --- | --- | --- | --- |
| Task | Measure | *n* | *M* | *SD* | Med | Min | Max | Centile | *n* | *M* | *SD* | Med | Min | Max | Centile |
| Picture Naming | Accuracy | 84 |  |  | 4 | 3 | 4 | 3 | 232 |  |  | 4 | 1 | 4 | 3 |
| Semantics | Accuracy | 84 |  |  | 4 | 3 | 4 | 3 | 232 |  |  | 4 | 2 | 4 | 3 |
| Orientation | Accuracy | 84 |  |  | 4 | 2 | 4 | 3 | 232 |  |  | 4 | 3 | 4 | 3 |
| Word recall | Encoding 1 | 84 |  |  | 4 | 1 | 5 | 2 | 232 |  |  | 5 | 2 | 5 | 3 |
|  | Encoding 2 accuracy | 84 |  |  | 5 | 0 | 5 | 4 | 232 |  |  | 5 | 0 | 5 | 4 |
|  | Delayed Recall accuracy | 84 |  |  | 4 | 0 | 5 | 1 | 232 |  |  | 5 | 0 | 5 | 2 |
|  | Delayed Recall and recognition | 84 |  |  | 5 | 4 | 5 | 4 | 232 |  |  | 5 | 3 | 5 | 4 |
| Episodic Recognition | Episodic Recognition accuracy | 84 |  |  | 3 | 2 | 4 | 3 | 231 |  |  | 4 | 1 | 4 | 3 |
| Trails | Processing speed | 84 | 34.27 | 18.71 |  | 15.81 | 98.34 |  | 179* | 35.35 | 19.91 |  | 14 | 143 |  |
|  | Executive Score | 84 | 76.30 | 29.17 |  | 0 | 100 |  | 232 | 83.78 | 23.11 |  | 0 | 100 |  |
| Rule Finding | Accuracy | 84 | 23.96 | 7.03 |  | 5 | 40 |  | 232 | 27.88 | 8.33 |  | 3 | 43 |  |
|  | Number of rules learned | 84 | 2.57 | 1.20 |  | 0 | 5 |  | 232 | 3.15 | 1.33 |  | 0 | 5 |  |
| Figure copy | Accuracy | 82 | 53.88 | 6.83 |  | 33 | 60 |  | 176* | 56.31 | 6.27 |  | 19 | 60 |  |
|  | Figure recall accuracy | 83 | 39.04 | 12.05 |  | 13 | 59 |  | 228 | 45.29 | 9.52 |  | 15 | 60 |  |
| Cancellation | Accuracy | 84 | 29.66 | .67 |  | 27 | 30 |  | 231 | 29.68 | .62 |  | 27 | 30 |  |
|  | False positives | 84 | .08 | .27 |  | 0 | 1 |  | 232 | .07 | .25 |  | 0 | 1 |  |
| Invisible cancellation | Accuracy | 84 | 28.32 | 1.69 |  | 23 | 30 |  | 231 | 28.49 | 1.87 |  | 13 | 30 |  |
|  | Correct revisits | 84 | .64 | 1.60 |  | 0 | 10 |  | 231 | .37 | .98 |  | 0 | 7 |  |

Note: Means and *SD*s reported only for tasks with sufficient range in values. We proposed to use Z-score based impairments greater than 2 SDs from the mean for measures we use mean and *SD* for. Measures with small ranges of possible scores are reported as median, min, max, and centiles. 5^th^ centiles are chosen from determining cut offs for impairment for low range tasks. Asterisks reflect UK only norm data.

**
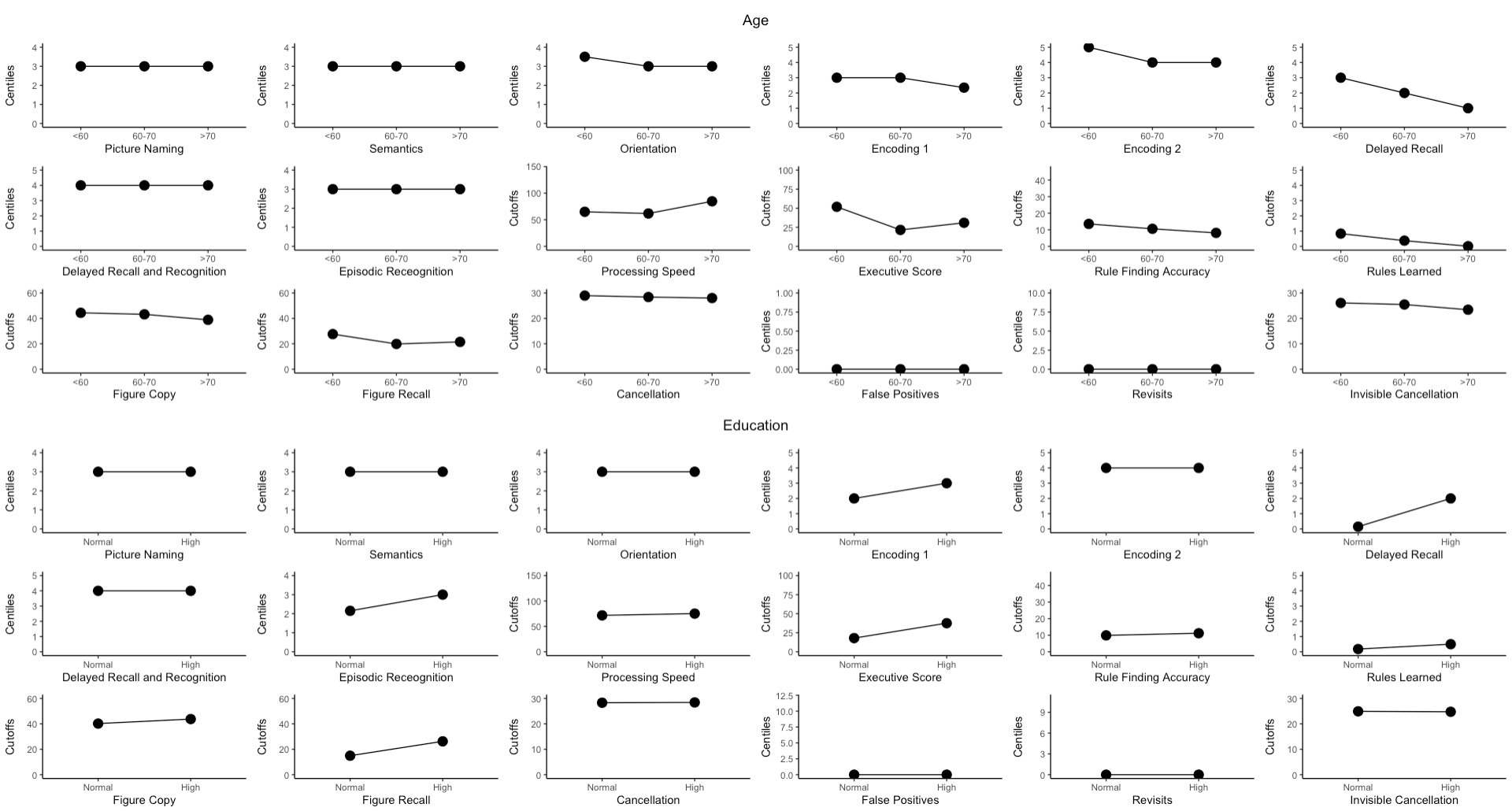
**

*Figure S5*. Illustrates the trend for each of the categories (age or education) per subtask on the OCS-plus for the cut offs, whether centile or 2*SD* based, to increase or decrease dependent on higher age or lower education specifically on some task. Figure created in RStudio (version 3.5.1^47^) using ggplot2^50^ (version 3.3.2)**,** figure available at <https://osf.io/9bd42/> under a CC-BY4.0 license.

**Test-Retest Analysis**

Table S8

*Wilcoxon signed rank test with continuity correction for test retest differences in OCS-Plus sub-tasks in 30 neurologically healthy adults from the UK (n=19) and Germany (n=11)*

| Measure | *M* time 1 | *M* time 2 | *p* | *r _effect size_* | *r _corr_* | *M RCI* | *Min RCI* | *Max RCI* | *> 90% < 95% CI* | *>95%* |
| --- | --- | --- | --- | --- | --- | --- | --- | --- | --- | --- |
| Picture Naming | 3.90 | 3.97 | .35 | 0 | .55^a^ | .49 | -.26 | 3.68 | 0 | 6.67 |
| Semantics | 3.93 | 3.80 | .13 | -.07 | .28 ^a^ | .67 | -2 | 2.61 | 0 | 20 |
| Orientation | 3.9 | 4 | .15 | 0 | - | .59 | -.33 | 2.95 | 0 | 10 |
| Encoding 1 | 4.47 | 4.67 | .22 | .05 | .45 | .72 | -2.60 | 2.13 | 0 | 10 |
| Encoding 2 | 5 | 4.93 | .35 | 0 | - | .49 | -3.68 | .26 | 0 | 6.67 |
| Delayed Recall | 3.60 | 3.67 | .63 | 0 | .17 ^a^ | .64 | -1.54 | 2.80 | 0 | 3.33 |
| Delayed Recall and Recognition | 4.17 | 4.43 | .24 | -.05 | .16 | .67 | -2.66 | 3.04 | 3.33 | 6.67 |
| Episodic Recognition | 3.60 | 3.67 | .63 | 0 | .17 | .64 | -1.54 | 2.80 | 0 | 3.33 |
| Rule Finding Accuracy | 27.73 | 28.67 | .45 | 0 | .43 ^a^ | .80 | -2.13 | 2.15 | 3.33 | 6.67 |
| Rules Learned | 3.43 | 3.70 | .39 | 0 | .32 | .78 | -1.58 | 1.90 | 10 | 0 |
| Trails Executive Score | 86.86 | 86.88 | .99 | .02 | -.13 | .73 | -1.97 | 2.69 | 3.33 | 6.67 |
| Trails Processing Speed | 24.18 | 23 | .50 | -.08 | .07 | .69 | -1.34 | 3.15 | 0 | 6.67 |
| Cancellation Accuracy | 29.80 | 29.77 | .78 | 0 | .32 | .53 | -1.97 | 2.11 | 0 | 23.33 |
| Cancellation False Positives | 0 | .03 | 1 | 0 | - | .35 | -018 | 5.29 | 0 | 3.33 |
| Invisible Cancellation Accuracy | 0 | 0 | .75 | .03 | .13 | .64 | -2.67 | 3.24 | 0 | 6.67 |
| Invisible Cancellation Revisits | .53 | .43 | .62 | -.41 | .42 | .57 | -3.28 | 2.37 | 0 | 10 |
| Figure Copy | 55.30 | 45.03 | .52 | -.51 | -.02 | .78 | -1.99 | 1.29 | 16.67 | 3.33 |
| Figure Recall | 40.33 | 36.57 | .79 | -.53 | -.07 | .74 | -2.07 | 1.67 | 13.33 | 3.33 |

*Note.* Oxford Cognitive Screen – Plus (OCS-Plus). *M*= mean. ‘*r _effect size_*’ = Wilcoxon signed rank test with continuity correction effect size. *r _corr_* = Kendall’s correlation between scores at time one and time two per task. RCI is reliable change index, that is, the value of reliable and significant change between testing sessions with values greater than 1.645 indicating a reliable increase in performance, and scores below -1.645 indicating reliable decrease in scores. Mean RCI is absolute, otherwise the mean is 0. CI = confidence interval. ‘*> 90% < 95% CI’* refers to the percentage of change scores between 90 and 95% CIs. ‘*>*95’ refers to percentage over 95% CI. Thirty neurologically healthy controls were retested across time, we analysed group-level performance using paired-samples Wilcoxon signed rank test with continuity correction. The correlation between time one and two could not be computed for Orientation, Encoding 2, or False positives, due to lack of variance. Mean retest interval was 320 days (*SD*=265.89, range=30-1182). ‘^a^’ refers to corrected correlations that are greater than 1 which suggests the measurement error is not randomly distributed, as such we do not report the corrected correlations here, these can be found in the open data/code.
